# Tumor-mesothelium HOXA11-PDGF BB/TGF β1-miR-181a-5p-Egr1 feedforward amplifier circuity propels mesothelial fibrosis and peritoneal metastasis of gastric cancer

**DOI:** 10.1038/s41388-023-02891-4

**Published:** 2023-11-21

**Authors:** Chao Wang, Jun Ji, Yangbing Jin, Ying Sun, Qu Cai, Jinling Jiang, Liting Guo, Chenfei Zhou, Jun Zhang

**Affiliations:** 1 Department of Oncology, Ruijin Hospital, Shanghai Jiao Tong University School of Medicine, No. 197 Ruijin er Road, Shanghai, 200025 China; 2 Shanghai Institute of Digestive Surgery, Ruijin Hospital, Shanghai Jiao Tong University School of Medicine, No. 197 Ruijin er Road, Shanghai, 200025 China

**Keywords:** Gastric cancer, Mechanisms of disease

## Abstract

A proportion of gastric cancer (GC) patients suffer from peritoneal metastasis (PM) in the late stage of tumor and these patients have a poor prognosis. To provide more care for GC patient with PM, a deeper exploration of the molecular characteristics of GC-PM is needed. Here we performed the in vitro and in vivo study to illustrate the effect of HOXA11 over-expressed GC cells on peritoneal mesothelial cells (HMrSV5), transcriptomics analyses of HMrSV5 cells co-cultured with HOXA11 over-expressed GC cells, counterparts or alone, cytokine array analyses of serum-free culture medium of HOXA11 over-expressed GC cells, we validated our findings through genetic manipulation of HMrSV5 cells and neutralizing antibodies targeting cytokines secreted by HOXA11 over-expressed GC cells in vitro, as well as utilized human peritoneal metastatic lesions to validate expression of potential targets. We identified that HOXA11 over-expressed GC cells strongly propelled mesothelial fibrosis in vivo and in vitro, and HOXA11 regulated paracrine and autocrine of PDGF BB and TGF β1 in GC cells to propel mesothelial fibrosis. Meanwhile, HOXA11 over-expressed GC cells drove PDGF BB and TGF β1 secretion to activate developmental-process related genes in HMrSV5 cells, including Egr1, which processes dependent on miR-181a-5p. Then, Egr1 could mediate peritoneal mesothelial fibrosis. Correspondingly, Egr1 over-expressed HMrSV5 cells supported migration and peritoneal dissemination of GC cells. Together our results suggest that a feedforward amplifier circuity governing GC cells and mesothelial cells in peritoneum contribute to peritoneal metastasis of GC cells.

## Introduction

The propensity of an organ to foster metastatic lesions is variable, with the lung, liver, ovarian, peritoneum, and bone being common metastatic sites in gastric cancer [1, 2]. Primary gastric cancers (GCs) tend to metastasize to particular organs, a relationship called organotropism, in contrast to hepatic or pulmonary metastasis, where GC cells invade the blood circulation or lymphatics system, direct invading into the peritoneal cavity is the most common manner for peritoneal metastasis (PM) [3, 4]. The reported risk factors for peritoneal seeding contain advanced tumor stage (T4), lymph node metastasis, histological subtype and positive peritoneal fluid cytology [3]. To construct a suitable microenvironment is one of the distinct biological properties during peritoneal dissemination, GC cells interact with other types of cells in the metastatic site [5], and the Paget’s “seed-and-soil theory” is a notion of the premetastatic niche where a microenvironment in a secondary organ devotes to the metastasis of a primary tumor [6].

The microenvironment contains every constituent of the malignant cancer other than the cancer cells themselves [7], especially, the tumor microenvironments (TME) in abdomen mainly included tumor cells, mesothelial cells, various types of immune cells, and different cytokines secreted by above types of cells [8]. Mesothelial cells through mesothelial to mesenchymal transition (MMT), were a source of cancer-associated fibroblasts (CAF) [8], noteworthily, High-metastatic disseminated gastric cancer cells strongly converted the surrounding stromal fibroblasts into the activated state, inducing the favorable microenvironments for metastasis [6].

Early growth response factor-1 (Egr1) serves as a conductor of the fibrogenic orchestra and aberrant expression of Egr1 leads to fibrosis [9]. Besides, emerging and increasing evidence has shown Egr1 is closely related with tumor progression in gastric cancer [10, 11].

Our team focused on the neoadjuvant intraperitoneal and systemic chemotherapy (NIPS) of advanced GC with peritoneal metastases [12, 13], and our prior study have found HOXA11 is an activator of PM in GC and HOXA11 could form a positive feedback loop with Stat3 to regulate adhesion, motility and apoptosis/anoikis-resistance phenotypes of gastric cancer cells [1]. Although, BBI608, could shrink the peritoneal disseminated tumor volume shaped by HOXA11 over-expressed GC cells, the remnant tumor was engulfed by mesothelium [1], BBI608 mainly targets the “seeds” (that is, cancer cells themselves), while effective antimetastatic responses could also be fulfilled by delivery of drugs that change the “soil” of the peritoneal tissue microenvironments [14], which inspired our desire to investigate the “soil” of TME in the peritoneum.

In our work, HOXA11 over-expressed GC cells could propel fibrosis of peritoneal mesothelial cells in vitro and in vivo, promoting us to hypothesize that HOXA11 might be a driver of Egr1 activation in HMrSV5 cells, thus, we try to illustrate whether HOXA11 triggers Egr1 in HMrSV5 cells, the function of Egr1 in HMrSV5 cells specifically in abetting migration and stem-cell like property of GC cells, and molecular mechanism that GC HOXA11 activates peritoneal mesothelium Egr1. This finding should be clarified before validating HOXA11 and fibrotic inhibitors in the clinic.

## Results

### HOXA11 over-expressed gastric cancer cells strongly propelled mesothelial fibrosis in vivo and in vitro

**Fig. 1:**
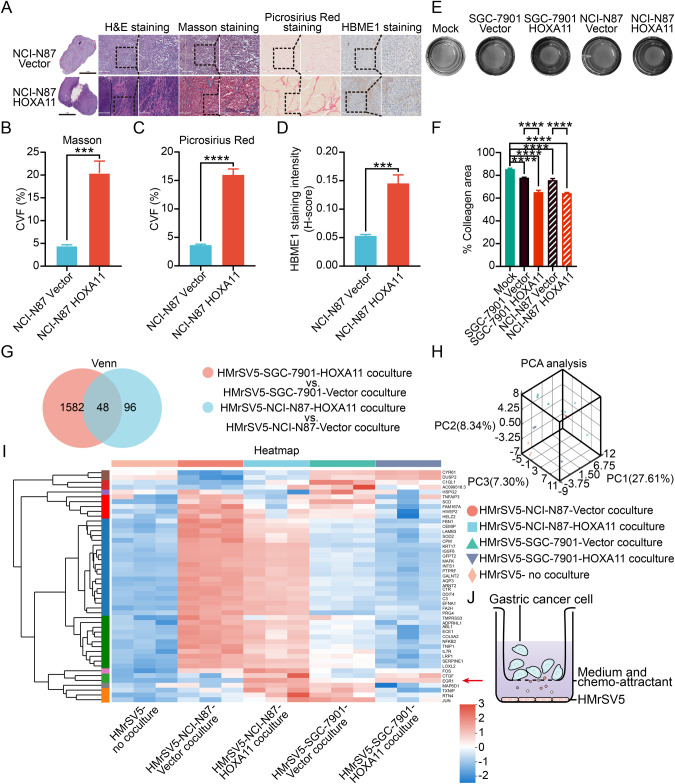
HOXA11 which expressed in gastric cancer cells could modulate the fibrosis of peritoneal mesothelial cells. **A** H&E images of peritoneal metastatic foci sections derived from BALB/c mice of NCI-N87-Vecotr and NCI-N87-HOXA11 gastric cancer cells groups, fibrosis was evaluated by Masson and Picrosirius Red staining of collagen, and HBME1 was the marker of peritoneal mesothelial cells. The scale bar, from left to right, 1 mm, 20× magnification; 200 μm, 100× magnification; 100 μm, 200× magnification. Representative pictures of *n* = 3 independent experiments. **B** Collagen volume fraction (CVF) was measured by Masson staining, and the quantified results were presented. Bar charts shown data as mean values ± SD over *n* = 3 biologically independent samples. ***P < 0.001. Statistical significance was assessed with two-tailed Mann-Whitney *U*-test. **C** Collagen volume fraction (CVF) was measured by Picrosirius Red staining, and the quantified results were presented. Bar charts shown data as mean values ± SD over *n* = 3 biologically independent samples. ****P < 0.0001. Statistical significance was assessed with two-tailed Student *t* test. **D** Statistical analysis of HBME1 staining intensity (H-score) in both groups. Bar charts shown data as mean values ± SD over *n* = 3 biologically independent samples. ***P < 0.001. Statistical significance was assessed with two-tailed Student *t* test. **E** The representative images of gel contraction assay shown the effect of HOXA11 exotic-expressed gastric cancer cells on the ability of HMrSV5 cells to contract type I collagen in vitro, as the gastric cancer cells added on the transwell chamber (0.4 μm). **F** Quantification of the areas were measured using ImageJ. Bar charts shown data as mean values ± SD over *n* = 3 biologically independent samples. ****P < 0.0001. Statistical significance was assessed with one-way ANOVA with Tukey’s HSD test. **G** The Venn diagram summarized the shared genes which is differentially expressed in HMrSV5 cells regulated by HOXA11 and counterparts in NCI-N87 and SGC-7901 gastric cancer cells. **H** The principal component analysis (PCA) of mRNA expression profiling data in HMrSV5 cells regulated by HOXA11 and counterparts in NCI-N87 and SGC-7901 gastric cancer cells. Axes depict principal component 1 (PC 1), principal component 2 (PC 2), and principal component 3 (PC 3). **I** Heatmap representation of the expression level of the 48 genes in HMrSV5 cells regulated by HOXA11 and counterparts in NCI-N87 and SGC-7901 gastric cancer cells and itself. Red arrow stands for Egr1. **J** The diagram illustrating the design of co-culture experiment.

Based on our previous work showing that HOXA11 overexpression could accelerate peritoneal dissemination of gastric cancer (GC) cells [1], we examined peritoneal metastatic lesions formed by HOXA11 over-expressed GC cells and its counterparts to determine whether HOXA11 has the ability to form a favorable microenvironment for peritoneal metastasis of GC cells. Firstly, the mesothelial fibrosis in lesions was evaluated by immunohistochemistry staining for Masson and Picrosirius Red staining of collagen and HBME1, a hallmark of peritoneal mesothelial cells (Fig. 1A). Hematoxylin-eosin (H&E) staining clearly separated tumor fraction and stromal ones, and collagen volume fraction (CVF) was significantly higher in the group of NCI-N87-HOXA11 compared with that in the control ones (Fig. 1B, C). Likewise, the expression level of HBME1 increased obviously in the group of NCI-N87-HOXA11 compared with that in the control ones, and the HBME1^+^ positive regions were almost in accordance with the Masson^+^ and Picrosirius Red^+^ positive area (Fig. 1D). To further evaluate the mesothelial fibrosis capacity of HOXA11, the gel contraction assay was performed. Comparing with the HMrSV5 in mono-culture cells and HMrSV5 in co-cultured with GC cells infected with blank virus, HOXA11 over-expressed GC cells significantly promoted the collagen-contracting ability of HMrSV5 cell in vitro (Fig. 1E, F). These data suggest that HOXA11 over-expressed GC cells strongly affect the surrounding peritoneal mesothelial cells and convert them into fibrosis status, presumably generating a favorable microenvironment.

### HOXA11 over-expressed GC cells induced developmental-process related genes in HMrSV5 cells

**Fig. 2:**
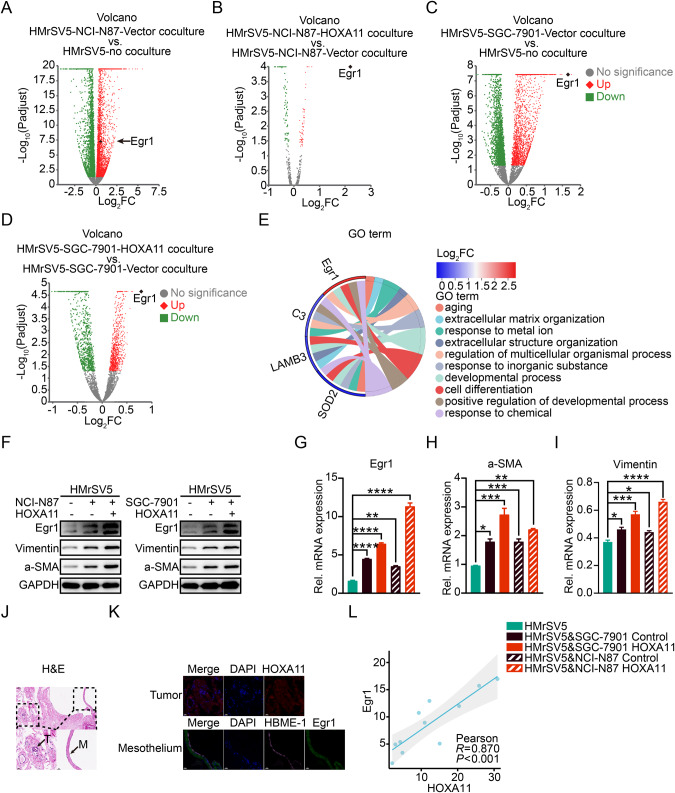
Egr1 was upregulated in HMrSV5 cells regulated by HOXA11 and counterparts in NCI-N87 and SGC-7901 GC cells, and peritoneal metastatic lesion of GC patient. **A** Volcano plots shown significantly changed genes in HMrSV5 cells co-cultured with NCI-N87 Vector or not with fold-change > 2 (labeled in red) or <0.5 (labeled in green) and *p* value <0.05. **B** Volcano plots shown significantly changed genes in HMrSV5 cells co-cultured with NCI-N87 HOXA11 or NCI-N87 Vector with fold-change > 2 (labeled in red) or <0.5 (labeled in green) and p value <0.05. **C** Volcano plots shown significantly changed genes in HMrSV5 cells co-cultured with SGC-7901 Vector or not with fold-change > 2 (labeled in red) or <0.5 (labeled in green) and *p* value <0.05. **D** Volcano plots shown significantly changed genes in HMrSV5 cells co-cultured with SGC-7901 HOXA11 or SGC-7901 Vector with fold-change > 2 (labeled in red) or <0.5 (labeled in green) and *p* value <0.05. **E** Chordal graph shown the functional analysis of shared up-regulated and down-regulated genes in HMrSV5 cells regulated by HOXA11 and counterparts in NCI-N87 and SGC-7901 GC cells by GO enrichment. **F** Expression of Egr1, Vimentin and α-SMA in indicated cells were analyzed using western blot, and GAPDH was applied as a loading control. **G** Expression of Egr1 in indicated cells were analyzed using qRT-PCR. Bar charts shown data as mean values ± SD over *n* = 3 biologically independent samples. **P < 0.01; ****P < 0.0001. Statistical significance was assessed with one-way ANOVA with Tukey’s HSD test. **H** Expression of α-SMA in indicated cells were analyzed using qRT-PCR. Bar charts shown data as mean values ± SD over *n* = 3 biologically independent samples. *P < 0.05; **P < 0.01; ***P < 0.001. Statistical significance was assessed with one-way ANOVA with Tukey’s HSD test. **I** Expression of Vimentin in indicated cells were analyzed using qRT-PCR. Bar charts shown data as mean values ± SD over *n* = 3 biologically independent samples. *P < 0.05; ***P < 0.001; ****P < 0.0001. Statistical significance was assessed with one-way ANOVA with Tukey’s HSD test. **J** H&E images of peritoneal metastatic foci sections derived from advanced gastric cancer patients. The scale bar, from upper to bottom, 50 μm, 200× magnification; 20 μm, 400× magnification. Representative pictures of *n* = 11 independent experiments. M, mesothelial fraction with an arrowhead, T, tumor fraction with an arrowhead. **K** Immunofluorescence assay shown the expression of HOXA11 in gastric cancer cells and expression of Egr1 in mesothelial cells marked by HBME1 derived from peritoneal metastatic foci. The scale bar, 20 μm, 400× magnification. Representative pictures of *n* = 11 independent experiments. **L** The relationship between HOXA11 + GC cells (MFI, mean fluorescent intensity) and Egr1+mesothelial cells (MFI) in peritoneal foci from advanced gastric cancer patients were analyzed by scatter diagram. *N* = 11 biologically independent samples. Statistical significance was assessed with Pearson.

To illustrate the molecular mechanism of peritoneal mesothelial fibrosis induced by HOXA11 over-expressed GC cells, RNA-sequencing profiling of HMrSV5 cells in mono-culture, in co-cultured with HOXA11 over-expressed GC cells and its counterparts (Fig. 1G–J). 48 shared genes were identified between HMrSV5 cultured with NCI-N87-HOXA11 and its counterparts as well as HMrSV5 cultured with SGC-7901-HOXA11 and its counterparts (Fig. 1G, I). Principal component analysis (PCA) mapping with RNA-sequencing profiling data presented an obvious separation of samples into five groups corresponding to the mono-culture HMrSV5 cells, to co-cultured with NCI-N87-HOXA11 and its counterparts as well as to co-cultured with SGC-7901-HOXA11 and its counterparts (Fig. 1H). To determine the key molecule participating in the peritoneal mesothelial fibrosis, differentially expressed genes of HMrSV5 cells in mono-cultured and co-cultured with HOXA11 over-expressed GC cells or its counterparts were selected and the expression changes in the RNA-sequencing profiling data were validated by Western blotting, qRT-PCR, and immunohistochemistry assays. Intriguingly, among the differential expressed genes, the upregulation of Egr1 was identified in the HMrSV5 cells co-cultured with NCI-N87-HOXA11 or SGC-7901-HOXA11 cells compared with co-cultured with its counterparts and the ones co-cultured with NCI-N87-Vector or SGC-7901-Vector cells compared with the mono-cultured (Fig. 2A–D). Egr1 was also involved into the “regulation of multicellular organismal process”, “developmental process”, “cell differentiation”, “positive regulation of developmental process” and “response to chemical” identified by GO analysis (Fig. 2E). The protein and mRNA level of Egr1 were also upregulated in HMrSV5 cells co-cultured with HOXA11 over-expressed GC cells, and the expression level of Vimentin and α-SMA, two of classical hallmarks of fibrosis, increased significantly in the HMrSV5 cells co-cultured with HOXA11 over-expressed GC cells compared with that in mono-cultured, and even the ones co-cultured with its counterparts (Fig. 2F–I), in contrast, knockdown of HOXA11 exhibited the opposite tendency of HMrSV5 cells (Fig. S2i). To further investigate the expression of Egr1 and HOXA11 in peritoneal metastatic lesions of GC patients, the immunohistochemistry and immunofluorescence experiments were conducted, as shown in Fig. 2J, K, the HBME1 and Egr1 were strongly expressed in the omentum, while HOXA11 expression was noticeably high in the tumor tissues. Furthermore, a strong positive correlation was found between the expression of HOXA11 and that of Egr1 (Pearson correlation = 0.870, *P* < 0.001) (Fig. 2L). Thus, our data indicated that Egr1 is key molecules involved in the peritoneal mesothelial fibrosis promoted by HOXA11 over-expressed GC cells.

### Peritoneal mesothelial cells supported migration and peritoneal dissemination of GC cells

**Fig. 3:**
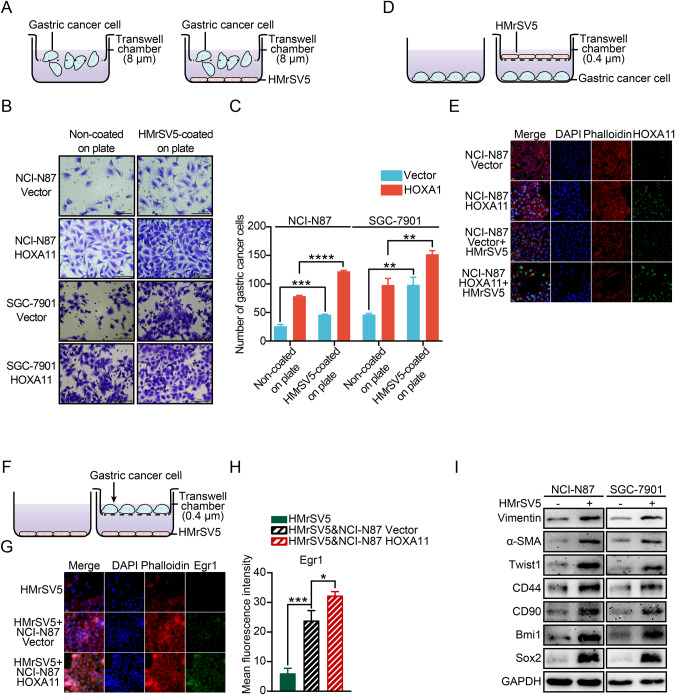
The effect of HMrSV5 cells on gastric cancer cells under co-cultured condition. **A** The diagram illustrating the design of chemotaxis assay. **B** Representative images of chemotaxis assay. The scale bar, 100 μm, 200× magnification. **C** Statistical analysis of number of migratory cells. Bar charts shown data as mean values ± SD over *n* = 3 biologically independent samples. **P < 0.01; ***P < 0.001, ****P < 0.0001. Statistical significance was assessed with two-tailed Student *t* test. **D** The diagram illustrating the design of co-culturing model. **E** Representative immunofluorescence images of HOXA11 (green) and Phalloidin (red) in HOXA11-overexpressed NCI-N87 cells and its counterparts when co-cultured with HMrSV5 cells or alone. The scale bar, 20 μm, 400× magnification. **F** The diagram illustrating the design of co-culturing model. **G** Representative immunofluorescence images of Egr1 (green) and Phalloidin (red) in HMrSV5 cells when co-cultured with HOXA11-overexpressed NCI-N87 cells and its counterparts or alone. The scale bar, 20 μm, 400× magnification. **H** Statistical analysis of Egr1 + HMrSV5 cells (MFI). Bar charts shown data as mean values ± SD over *n* = 3 biologically independent samples. *P < 0.05; ***P < 0.001. Statistical significance was assessed with one-way ANOVA with Tukey’s HSD test. **I** The protein expression of Vimentin, α-SMA, Twist1, CD44, CD90, Bmi1 and Sox2 in NCI-N87 and SGC-7901 co-cultured with HMrSV5 cells or not were analyzed using western blot with the indicated antibodies. GAPDH was included as a loading control.

**Fig. 4:**
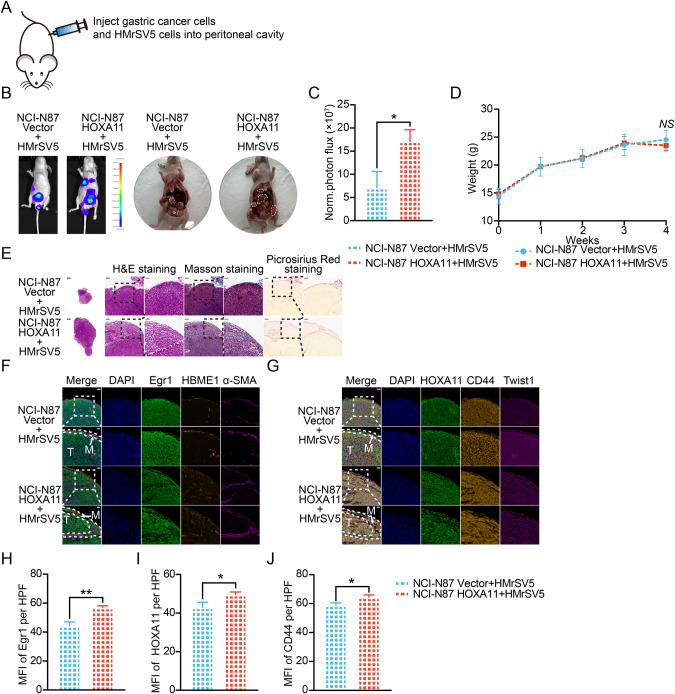
Peritoneal mesothelial cells accelerated the tumorigenesis of gastric cancer cells in the peritoneum. **A** The diagram illustrating the design of in vivo assay. **B** HMrSV5 cells ameliorated peritoneal metastasis of HOXA11 -overexpressed NCI-N87 cells in BALB/c mice. Tumor in peritoneum were measured both in situ and after laparotomy. **C** Statistical analysis of the bioluminescence in peritoneal foci of both groups. Bar charts shown data as mean values ± SD over *n* = 3 biologically independent samples. *P < 0.05. Statistical significance was assessed with two-tailed Student *t* test. **D** Body weights were measured weekly, and there was no significant decrease of body weight in co-cultured group compared with littermate. Bar charts shown data as mean values ± SD over *n* = 3 biologically independent samples. NS, no significance. Statistical significance was assessed with two-tailed Student *t* test. **E** Representative image of H&E-stained sections from peritoneal foci and fibrosis was evaluated by Masson and Picrosirius Red staining of collagen. The scale bar, from left to right, 500 μm, 20× magnification; 100 μm, 100× magnification; 50 μm, 200× magnification. Representative pictures of *n* = 3 independent experiments. **F** Representative immunofluorescence images of Egr1 + , α-SMA and HBME1+ mesothelial cells in peritoneal foci from co-cultured groups. The scale bar, from upper to bottom, 50 μm, 200× magnification; 20 μm, 400× magnification. M, mesothelial fraction in the area of dot line, T, tumor fraction outside the area of dot line. **G** Representative immunofluorescence images of Twist1 + , CD44+ and HOXA11 + GC cells in peritoneal foci from co-cultured groups. The scale bar, from upper to bottom, 50 μm, 200× magnification; 20 μm, 400× magnification. M, mesothelial fraction in the area of dot line, T, tumor fraction outside the area of dot line. **H**–**J** Statistical analysis of Egr1 + HMrSV5 cells, HOXA11 + GC cells and CD44 + GC cells (MFI) in peritoneal foci from co-cultured groups. Bar charts shown data as mean values ± SD over *n* = 3 biologically independent samples. *P < 0.05; **P < 0.01. Statistical significance was assessed with two-tailed Student *t* test.

Human peritoneal mesothelial cells (HPMCs), as a main origin of myofibroblasts in GC microenvironment, offer a suitable environment for the dissemination of GC [15, 16]. To further explore the function of HMrSV5 cells in this field, we cocultured HOXA11 over-expressed GC cells or its counterparts with HMrSV5 cells, and transwell chemotaxis experiment was conducted to confirm that HMrSV5 cells could enhance migration of GC cells, particularly in the group where HOXA11 is overexpressed (Fig. 3A–C), notably, HMrSV5 cells significantly impelled the migration of GC cells with endogenous high HOXA11 expression (MGC-803) (*P* < 0.0001), and slightly enhanced the migration of MGC-803 cells wherein HOXA11 had been knocked down (*P* < 0.05; Fig. S2J, K). Our previous study found that GC cells with forced HOXA11 expression could experience the epithelial-mesenchymal transition (EMT) [1], interestingly, the co-cultured HMrSV5 cells instigated morphological changes in NCI-N87 Vector cells, resulting in leading-trailing mesenchymal morphology with increased lamellipodia formation, this differed significantly from the cobble-stone-like appearance observed in monocultured NCI-N87 Vector cells. In addition, the co-cultured HMrSV5 cells induced even more lamellipodia formation in NCI-N87 HOXA11 cells which had undergone EMT (Fig. 3D, E). Conversely, HOXA11 could facilitate the process of MMT in HMrSV5 cells, increasing both lamellipodia formation and the expression of Egr1 (Fig. 3F–H). Western blotting analysis revealed that mesenchymal markers (Vimentin, α-SMA, and Twist1), stem cell-like property markers (CD44, CD90 and Bmi1) and pluripotency marker (Sox2) were increased in GC cells when co-cultured with HMrSV5 cells (Fig. 3I). To perform proof-of principle in vivo study, NCI-N87-Vector or NCI-N87-HOXA11 cells mixed with HMrSV5 cells were transplanted into peritoneal cavity of BALB/c mice (Fig. 4A), cells stably expressed luciferase and peritoneal dissemination of tumor were observed by an in vivo imaging system (IVIS) after four weeks. Using the IVIS, we found increased bioluminescent signal intensities in the peritoneal cavities of the mice injected with NCI-N87-HOXA11 cells and HMrSV5 cells (*P* < 0.05) (Fig. 4B, C), upon dissection, the NCI-N87-HOXA11 cells and HMrSV5 cells transplanted mice exhibited a significantly higher metastatic burden in the peritoneal cavity compared to the mice transplanted with NCI-N87-Vector and HMrSV5 cells (Fig. 4B), while, there was no difference between the above two groups in terms of body weight (Fig. 4D). Moreover, H&E, Masson and Picrosirius Red staining have clearly separated tumor fraction and stromal ones (Fig. 4E), and we clearly observed Egr1, α-SMA&HBME1-positive mesothelial cells in the stromal fraction, the Egr1 positive mesothelial cells in stromal fraction were obviously increased in the mice injected with NCI-N87-HOXA11 cells and HMrSV5 cells in comparison to those of mice injected with NCI-N87-Vector cells and HMrSV5 cells (*P* < 0.01) (Fig. 4F, H). CD44 positive GC cells and HOXA11 positive GC cells in tumor fraction were significantly increased in the mice injected with NCI-N87-HOXA11 cells and HMrSV5 cells in comparison to those of mice injected with NCI-N87-Vector cells and HMrSV5 cells (*P* < 0.05) (Fig. 4G, I, J). Taken together, these findings indicated that HOXA11 spurs mesothelial fibrosis which could subsequently promote migration and peritoneal dissemination of GC cells and enhance the stem cell-like property of GC cells themselves.

### HOXA11 regulated the paracrine and autocrine of PDGF BB and TGF β1 in GC cells to propel mesothelial fibrosis

**Fig. 5:**
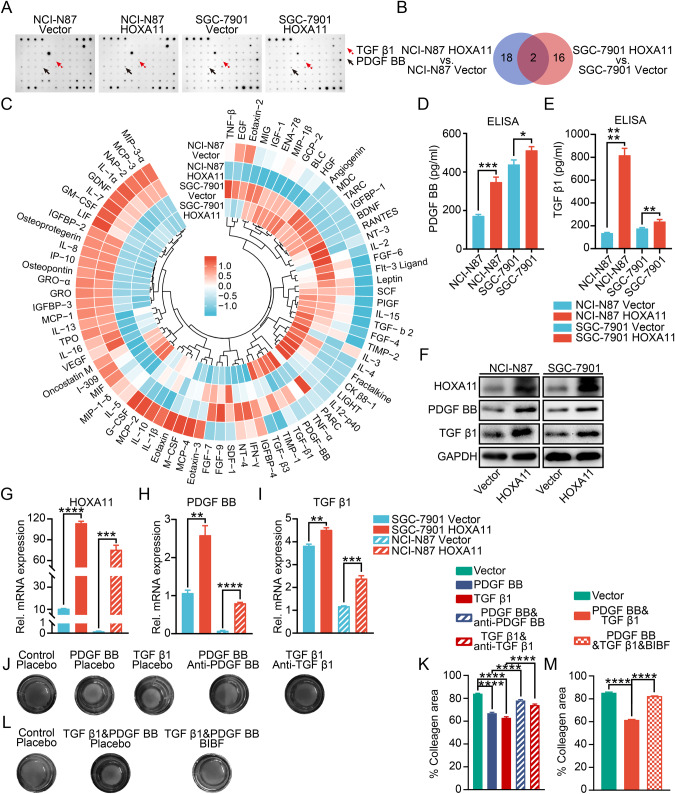
The paracrine effect of PDGF BB and TGF β1 released by HOXA11 overexpressed GC cells on peritoneal mesothelial cells. **A** Cytokine array shown altered abundance of chemokines/cytokines in serum-free cultured medium collected from NCI-N87 cells and SGC-7901 cells stably expressing HOXA11 or Vector. red arrow, TGF β1; black arrow, PDGF BB. **B** The Venn diagram summarized the shared chemokines/cytokines which is altered by HOXA11 in NCI-N87 and SGC-7901 GC cells. **C** The circus heatmap shown fold changes in protein levels of the altered chemokines/cytokines in NCI-N87 and SGC-7901 GC cells stably expressing HOXA11 or Vector. **D** The density of PDGF BB in serum-free cultured medium collected from NCI-N87 cells and SGC-7901 cells stably expressing HOXA11 or Vector were measured using ELISA assay. Bar charts shown data as mean values ± SD over *n* = 3 biologically independent samples. *P < 0.05; ***P < 0.001, Statistical significance was assessed with two-tailed Student *t* test. **E** The density of TGF β1 in serum-free cultured medium collected from NCI-N87 cells and SGC-7901 cells stably expressing HOXA11 or Vector were measured using ELISA assay. Bar charts shown data as mean values ± SD over *n* = 3 biologically independent samples. **P < 0.01; ****P < 0.0001, Statistical significance was assessed with two-tailed Mann-Whitney *U*-test, and two-tailed Student *t* test, respectively. **F** Representative immunoblots of Vector- and HOXA11-overexpressed NCI-N87 and SGC-7901 cell lysates blotted as indicated, GAPDH was included as a loading control. **G**–**I** qRT-PCR shown mRNA levels of the indicated genes in NCI-N87 cells and SGC-7901 cells stably expressing HOXA11 or Vector. Bar charts shown data as mean values ± SD over *n* = 3 biologically independent samples. **P < 0.01; ***P < 0.001; ****P < 0.0001, Statistical significance was assessed with two-tailed Mann-Whitney *U*-test, and two-tailed Student *t* test, respectively. **J** Representative images of gel contraction assay shown the effect of PDGF BB, TGF β1, neutralizing antibody of PDGF BB, and neutralizing antibody of TGF β1 on the ability of HMrSV5 cells to contract type I collagen in vitro. **K** Quantification of gel contraction assay from experiments. Bar charts shown data as mean values ± SD over *n* = 3 biologically independent samples. ****P < 0.0001, Statistical significance was assessed with one-way ANOVA with Tukey’s HSD test. **L** Representative images of gel contraction assay shown the effect of both PDGF BB and TGF β1, and BIBF on the ability of HMrSV5 cells to contract type I collagen in vitro. **M** Quantification of gel contraction assay from experiments. Bar charts shown data as mean values ± SD over *n* = 3 biologically independent samples. ****P < 0.0001, Statistical significance was assessed with one-way ANOVA with Tukey’s HSD test.

**Fig. 6:**
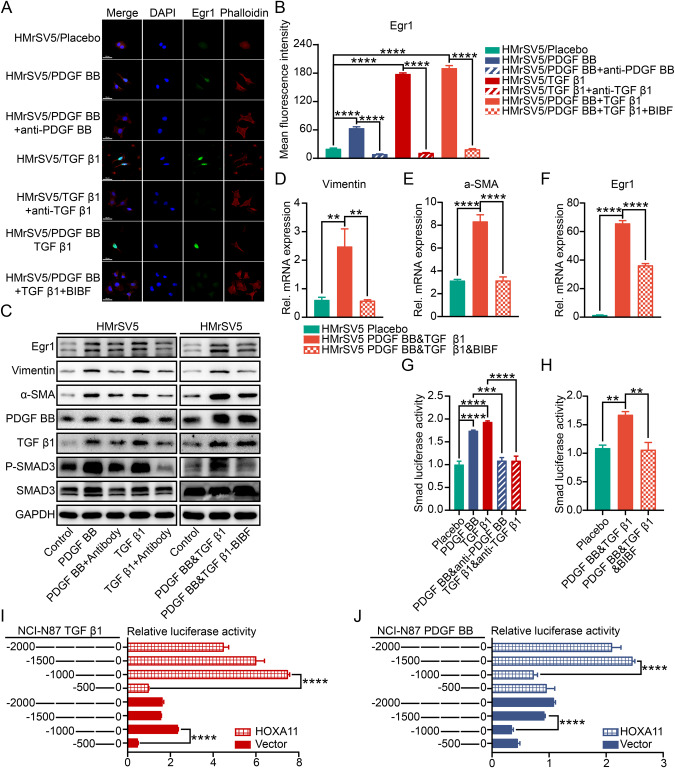
Paracrine PDGF BB and TGF β1 activated fibrosis in peritoneal mesothelial cells. **A** Representative immunofluorescence images of Egr1 (green) and Phalloidin (red) in HMrSV5 cells after the addition of PDGF BB, TGF β1, neutralizing antibody of PDGF BB, neutralizing antibody of TGF β1 or BIBF. The scale bar, 50 μm, 400× magnification. **B** Statistical analysis of Egr1 + HMrSV5 cells (MFI, mean fluorescent intensity). Bar charts shown data as mean values ± SD over *n* = 3 biologically independent samples. ****P < 0.0001. Statistical significance was assessed with one-way ANOVA with Tukey’s HSD test. **C** Representative immunoblots of PDGF BB, TGF β1, neutralizing antibody of PDGF BB, neutralizing antibody of TGF β1 or BIBF treated HMrSV5 cell lysates blotted as indicated, GAPDH was included as a loading control. **D**-**F** qRT-PCR shown mRNA levels of the indicated genes in HMrSV5 cells after the addition of PDGF BB, TGF β1, and BIBF or Placebo. Bar charts shown data as mean values ± SD over *n* = 3 biologically independent samples. **P < 0.01; ****P < 0.0001, Statistical significance was assessed with one-way ANOVA with Tukey’s HSD test. **G** Smad luciferase reporter activity in HMrSV5 cells after the addition of PDGF BB, TGF β1, neutralizing antibody of PDGF BB or neutralizing antibody of TGF β1. Bar charts shown data as mean values ± SD over *n* = 3 biologically independent samples. ***P < 0.001; ****P < 0.0001. Statistical significance was assessed with one-way ANOVA with Tukey’s HSD test. **H** Smad luciferase reporter activity in HMrSV5 cells after the addition of PDGF BB and TGF β1, and BIBF or Placebo. Bar charts shown data as mean values ± SD over *n* = 3 biologically independent samples. **P < 0.01. Statistical significance was assessed with one-way ANOVA with Tukey’s HSD test. **I**, **J** TGF β1 and PDGF BB luciferase reporter activity in NCI-N87 cells stably expressing HOXA11 or Vector with altered promoter zones. Bar charts shown data as mean values ± SD over *n* = 3 biologically independent samples. ****P < 0.0001, Statistical significance was assessed with one-way ANOVA with Tukey’s HSD test.

**Fig. 7:**
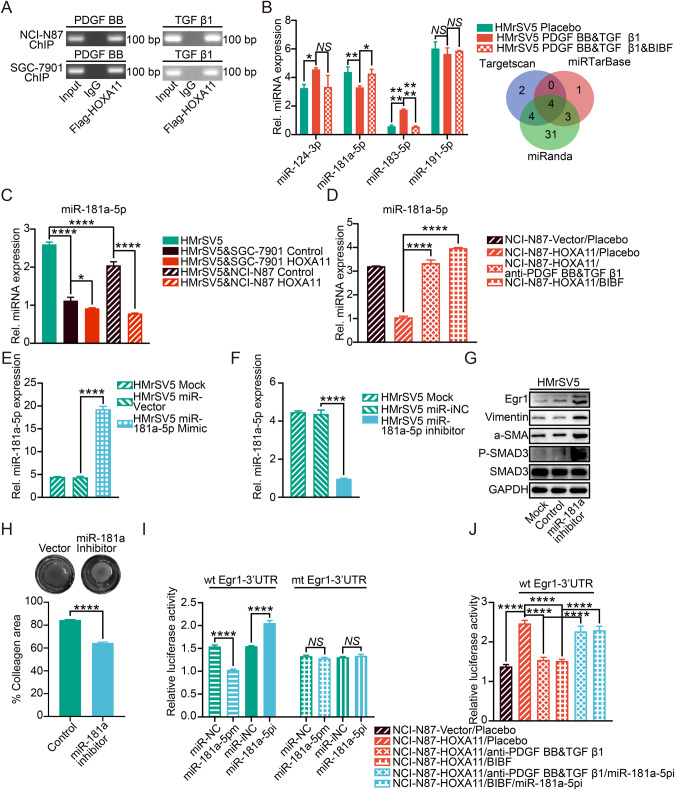
HOXA11 positively modulated the expression of Egr1 via inhibiting miR-181a-5p in HMrSV5 cells. **A** The ChIP-qPCR experiments were executed to assess whether transcription factor HOXA11 could bind on the promoter region of PDGF BB and TGF β1 in NCI-N87 and SGC-7901 cells. IgG was utilized as a negative control. **B** qRT-PCR shown expression levels of the indicated microRNA in HMrSV5 cells after the addition of PDGF BB, TGF β1, and BIBF or Placebo. Bar charts shown data as mean values ± SD over *n* = 3 biologically independent samples. *P < 0.05; **P < 0.01; ****P < 0.0001, NS, no significance; Statistical significance was assessed with one-way ANOVA with Tukey’s HSD test. the Venn diagram summarized the common microRNAs which target gene is Egr1 in Targetscan, miRTarBase and miRanda database. **C** qRT-PCR analysis of the expression level of miR-181a-5p in HMrSV5 cells regulated by HOXA11 and counterparts in NCI-N87 and SGC-7901 gastric cancer cells and itself. Bar charts shown data as mean values ± SD over *n* = 3 biologically independent samples. *P < 0.05; ****P < 0.0001; Statistical significance was assessed with one-way ANOVA with Tukey’s HSD test. **D** qRT-PCR analysis of the expression level of miR-181a-5p in HMrSV5 cells regulated by co-cultured HOXA11 over-expressed gastric cancer cells upon neutralizing PDGF BB and TGF β1 or adding BIBF. The result was normalized to U6 small nuclear RNA. Bar charts shown data as mean values ± SD over *n* = 3 biologically independent samples. ****P < 0.0001; Statistical significance was assessed with one-way ANOVA with Tukey’s HSD test. **E** qRT-PCR analysis of the expression level of miR-181a-5p in HMrSV5 cells transfected with miR-181a-5p mimics or Vector. The result was normalized to U6 small nuclear RNA. Bar charts shown data as mean values ± SD over *n* = 3 biologically independent samples. ****P < 0.0001; Statistical significance was assessed with one-way ANOVA with Tukey’s HSD test. **F** qRT-PCR analysis of the expression level of miR-181a-5p in HMrSV5 cells transfected with miR-181a-5p inhibitor or Control. The result was normalized to U6 small nuclear RNA. Bar charts shown data as mean values ± SD over *n* = 3 biologically independent samples. ****P < 0.0001; Statistical significance was assessed with one-way ANOVA with Tukey’s HSD test. **G** Representative immunoblots of Mock, Control- and miR-181a-5p inhibitor transfected HMrSV5 cell lysates blotted as indicated, GAPDH was included as a loading control. **H** Representative images of gel contraction assay shown the effect of miR-181a-5p inhibitor on the ability of HMrSV5 cells to contract type I collagen in vitro (upper). Quantification of gel contraction assay from above experiment. Bar charts shown data as mean values ± SD over *n* = 3 biologically independent samples. ****P < 0.0001, Statistical significance was assessed with two-tailed Student *t* test. **I** Luciferase assay of HMrSV5 cells co-transfected with miR-181a-5p mimics or inhibitors and the wild type- and mutant type- luciferase reporter. **J** The Luciferase assay of wild-type 3’UTR region of Egr1 in HMrSV5 cells regulated by GC cells-peritoneal mesothelial cells HOXA11-PDGF BB/TGF β1-miR-181a-5p feedforward amplifier circuitry. Bar charts shown data as mean values ± SD over *n* = 3 biologically independent samples. ****P < 0.0001; NS, no significance. Statistical significance was assessed with two-tailed Student *t* test (**I**) or one-way ANOVA with Tukey’s HSD test (**J**). wt wild-type, mt mutant type, miR-NC miRNA negative control, miR-181a-5pm miR-181a-5p mimics, miR-iNC miRNA inhibitor negative control, miR-181a-5pi miR-181a-5p inhibitor.

Tumor secreted cytokines and chemokines into both proximal surrounding cells and peritoneal cavity, thereby promoting the inoculation of a tumor microenvironment that in turn adapts peritoneal dissemination of tumor [17]. To illustrate the role of HOXA11 in this field, we did cytokine array analysis in culture media obtained from Vector or HOXA11 over-expressed GC cells. Using densitometry, we realized that two shared cytokines (PDGF BB and TGF β1) were upregulated in the culture media from HOXA11 over-expressed GC cells (Fig. 5A–C). We validated these findings by qRT-PCR and Western blotting of cell lysates of GC cells and ELISA of cellular medium (CM) of GC cells, Intriguingly, the mRNA and protein level of PDGF BB and TGF β1 were upregulated by HOXA11 in GC cells and similar findings were seen in CM of GC cells by ELISA assay (Fig. 5D–I), knockdown of HOXA11 in MGC803 cells exhibited an opposite effect (Fig. S2a–f). To further clarify function of these cytokines, GO analysis and KEGG pathway analysis were executed using 36 selected cytokines whose expression was significantly different in CM of HOXA11 over-expressed GC cells (Fig. S1a), “cytokine-cytokine receptor interaction” was one of the most significant alterations of KEGG pathways. As emerging and accumulating evidence had found that cytokines are the key cues for illustrating the molecular basis underlying the intercellular communications in the tumor microenvironment [18, 19], we supposed that PDGF BB and TGF β1 which released by HOXA11 over-expressed GC cells confer the mesothelial fibrosis in the co-culture system. To test this hypothesis, addition of PDGF BB and TGF β1 could significantly promote the collagen-contracting ability of HMrSV5 cell, respectively. Meanwhile, addition of anti-human PDGF BB and TGF β1 neutralizing antibodies could effectively block the collagen-contracting ability inspired by PDGF BB and TGF β1, respectively (Fig. 5J, K), the presence of PDGF BB receptor inhibitor BIBF, which could also inhibit the function of TGF β1 [20, 21], also block the collagen-contracting ability enhanced by both of PDGF BB and TGF β1 (Fig. 5L, M). In the co-culture system, addition of anti-human PDGF BB and TGF β1 neutralizing antibodies or BIBF could obviously thwart the collagen-contracting ability propelled by HOXA11 over-expressed GC cells (Fig. S1b). In accordance with the results of the gel contraction assay, the HMrSV5 cells initially had a cobblestone shape, they became spindle-shaped 24 h after the addition of PDGF BB and TGF β1, respectively, moreover, addition of anti-human PDGF BB, TGF β1 neutralizing antibodies or BIBF could reverse morphological changes caused by PDGF BB and TGF β1 (Fig. 6A). Interestingly, similar findings were seen with immunofluorescence, western blotting, and qRT-PCR analysis of Egr1 expression in HMrSV5 cells (Fig. 6B–F), TGF β1/Smad signaling pathway activation had been confirmed to participate into the pathogenesis of peritoneal fibrosis [22, 23], western blotting analysis revealed that exogenous PDGF BB and TGF β1 induced the phosphorylation of Smad3 in HMrSV5 cells and addition of anti-human PDGF BB, TGF β1 neutralizing antibodies or BIBF could reverse this tendency (Fig. 6C). To validate whether the inhibition of Smad3 phosphorylation affects downstream transcriptional responses regulated by PDGF BB and TGF β1, Smad luciferase reporter plasmid was used to perform transient transfection assays and exogenous PDGF BB and TGF β1 strongly prompted reporter activities, respectively, while these effects were antagonized by anti-human PDGF BB, TGF β1 neutralizing antibodies or BIBF (Fig. 6G, H). HOXA11 served as a transcription factor in the nucleus and played crucial roles in the tumorigenesis of various types of cancer [1, 24], and the above results confirmed that HOXA11 could upregulate the mRNA and protein levels of PDGF BB and TGF β1, thus, we hypothesized that HOXA11 enhances the transcriptional activity of PDGF BB and TGF β1 promoters. To test this hypothesis, the full length PDGF BB and TGF β1 promoters were cloned into a luciferase reporter plasmid respectively, and were then co-transfected with either HOXA11 plasmid or vector into NCI-N87 and SGC-7901 cells and the results showed a robust and positive response inspired by HOXA11 in both PDGF BB and TGF β1 promoters (Fig. 6I, J; Fig. S1c, d), while, knockdown of HOXA11 in MGC803 cells reversed this phenomenon (Fig. S2g, h), moreover, serial truncations of the PDGF BB and TGF β1 promoter based on the length of sequence were constructed to further refined the target sequence of the promoter. And the luciferase reporter experiment showed that deleting the region between −1500 and −1000 bases of PDGF BB promoter and the region between −1000 and −500 bases of TGF β1 promoter significantly abolished PDGF BB and TGF β1 promoter activity inspired by HOXA11, respectively (Fig. 6I, J; Fig. S1c, d). To determine whether HOXA11 can bind to the PDGF BB and TGF β1 promoters in intact cells, we supposed the binding sites of HOXA11 in the promoter zone of PDGF BB (the region between −1500 and −1000 bases) and TGF β1 (the region between −1000 and −500 bases) using the JASPAR database and performed ChIP-qPCR and the results confirmed that HOXA11 directly binds to the PDGF BB and TGF β1 promoters in NCI-N87 and SGC-7901 cells (Fig. 7A). These findings suggested that PDGF BB and TGF β1 were secreted from GC cells could activate fibrosis of HMrSV5 cells and transcriptionally regulated by HOXA11 to promote their fibrogenic function.

Besides, whether the secreted PDGF BB and TGF β1 could play a role in each other mutually in gastric cancer cells, to this end, ELISA assays were executed to compare the concentration of PDGF BB and TGF β1 in CM of GC cells after addition of PDGF BB and TGF β1 respectively, and we realized that PDGF BB and TGF β1 could elevate the concentration of TGF β1 and PDGF BB mutually (Fig. S1e, f). Consistent with our ELISA data, addition of PDGF BB and TGF β1 significantly enhanced the protein expression level of TGF β1 and PDGF BB mutually in GC cells by western bolts, meanwhile, PDGF BB and TGF β1 could also upregulate the expression level of Vimentin and α-SMA in GC cells, which might induce epithelial-mesenchymal transition in GC cells (Fig. S1g). Furthermore, the luciferase reporter assays have found that addition of PDGF BB and TGF β1 significantly stimulated PDGF BB and TGF β1 promoter activity mutually in GC cells (Fig. S1h). These results suggested that autocrine PDGF BB and TGF β1 activate PDGF BB and TGF β1 mutually in GC cells.

### GC HOXA11 drove PDGF BB and TGF β1 secretion to promote mesothelial fibrosis dependent on miR-181a-5p

To further explore the mechanism by which GC HOXA11 propel mesothelial fibrosis, and a prior study proved that miRNAs could induce degradation or translational repression of target gene mRNA by binding to target sites in the 3’UTR of mRNA [25]. For this purpose, we try to identify the miRNA which could play a role in the process of mesothelial fibrosis propelled by HOXA11 in HMrSV5 cells, analysis of public available algorithms (Targetscan, miRTarBase and miRanda) was performed to identify which miRNAs capable of targeting Egr1 3’UTR. miR-124-3p, miR-181a-5p, miR-183-5p and miR-191-5p were predicted to have potential target sites in 3’UTR of Egr1 gene (Fig. 7B), we transfected HMrSV5 cells with miR-124-3p, miR-181a-5p, miR-183-5p and miR-191-5p mimics respectively (Fig. 7E, Fig. S3a–c), and then western blotting experiments were performed to confirm that overexpression of miR-124-3p, miR-181a-5p, miR-183-5p or miR-191-5p could remarkably suppress the protein expression level of Egr1 in HMrSV5 cells, respectively (Fig. S3d). Furthermore, as illustrated by qRT-PCR analysis, addition of PDGF BB and TGF β1 could significantly upregulate the expression of miR-124-3p and miR-183-5p, whereas, downregulate the expression level of miR-181a-5p in HMrSV5 cells (Fig. 7B), while, addition of BIBF could obviously reverse the expression of miR-183-5p and miR-181a-5p regulated by PDGF BB and TGF β1 (Fig. 7B). Considering the expression of Egr1 is upregulated by PDGF BB and TGF β1 and expression of miRNA is negatively correlated with their target genes, we hypothesized that miR-181a-5p is participated into HOXA11-regulated mesothelial fibrosis in vitro, to validate this postulation, firstly, we sought to validate the expression level of miR-181a-5p in HMrSV5 cells when co-cultured with HOXA11-overexpressed GC cells, qRT-PCR analysis showed that HOXA11-overexpressed GC cells could significantly decrease the expression of miR-181a-5p and addition of anti-human PDGF BB and TGF β1 neutralizing antibodies or BIBF could remarkably thwart the inhibition of HOXA11-overexpressed GC cells (Fig. 7C, D). Secondly, addition of anti-human PDGF BB and TGF β1 neutralizing antibodies or BIBF could decrease the mRNA and protein expression level of α-SMA, Vimentin and Egr1 in HMrSV5 cells when co-cultured with HOXA11-overexpressed GC cells, in contrast, HMrSV5 cells transfected with inhibitor of miR-181a-5p revealed remarkably upregulated mRNA and protein expression level of α-SMA, Vimentin and Egr1 under the same condition (Fig. S3e–h). Moreover, inhibition of miR-181a-5p could not only upregulate the protein expression level of α-SMA, Vimentin and Egr1, but also enhance the phosphorylation of Smad3 in HMrSV5 cells (Fig. 7F, G), to further evaluate the mesothelial fibrosis capacity of miR-181a-5p, the gel contraction assay was performed and inhibition of miR-181a-5p promoted collagen-contracting ability of HMrSV5 cell in vitro (Fig. 7H). Thirdly, we constructed dual-luciferase reporters in 3’UTR region of Egr1 which contained wild-type or mutant binding site for miR-181a-5p and then these reporter constructs were co-transfected into HMrSV5 cells with miR-181a-5p mimics or miR-181a-5p inhibitor. Intriguingly, high expression of miR-181a-5p could decrease luciferase activity of wild-type groups and inhibition of miR-181a-5p enhanced the luciferase activity of wild-type groups, while there were no changes in the luciferase activity of mutant groups (Fig. 7I). Furthermore, the luciferase activity of wild-type 3’UTR region of Egr1 in HMrSV5 cells were activated when co-cultured with HOXA11 over-expressed GC cells, and then addition of anti-human PDGF BB and TGF β1 neutralizing antibodies or BIBF could obviously inhibit the effect of HOXA11 over-expressed GC cells, and the above tendency could be reversed once inhibiting the expression level of miR-181a-5p in HMrSV5 cells (Fig. 7J). In summary, HOXA11 over-expressed GC cells released PDGF BB and TGF β1 to promote mesothelial fibrosis dependent on miR-181a-5p.

### Egr1 mediated mesothelial fibrosis and prompted migration of GC cells

**Fig. 8:**
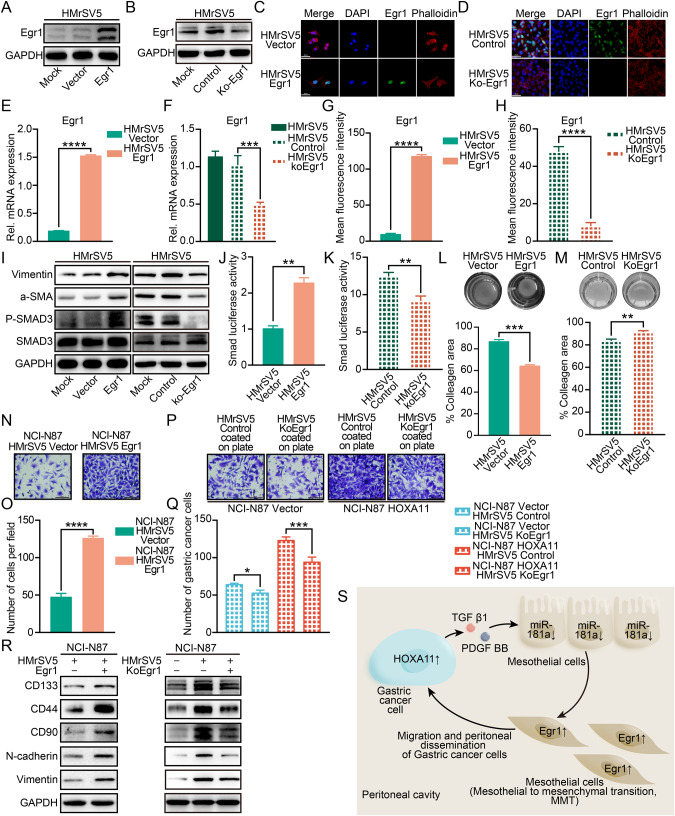
Egr1 accelerated the fibrosis and chemotaxis of peritoneal mesothelial cell itself and enhanced the expression of stemness-related markers in gastric cancer cells when co-cultured with peritoneal mesothelial cells which overexpressed Egr1. **A** Representative immunoblots of Mock, Vector- and Egr1- plasmid transfected HMrSV5 cell lysates blotted as indicated, GAPDH was included as a loading control. **B** Egr1 knockout in HMrSV5 cells were measured by immunoblot, GAPDH was included as a loading control. **C** Representative immunofluorescence images of Egr1 (green) and Phalloidin (red) in HMrSV5 cells stably expressing Egr1 or Vector. The scale bar, 50 μm, 400× magnification. **D** Representative immunofluorescence images of Egr1 (green) and Phalloidin (red) in HMrSV5-Control cells and HMrSV5-KoEgr1 cells. The scale bar, 50 μm, 400× magnification. **E** qRT-PCR shown mRNA levels of Egr1 in HMrSV5 cells stably expressing Egr1 or Vector. Bar charts shown data as mean values ± SD over *n* = 3 biologically independent samples. ****P < 0.0001, statistical significance was assessed with two-tailed Student *t* test. **F** The knockout efficiency of Egr1 was determined by qRT-PCR. Bar charts shown data as mean values ± SD over *n* = 3 biologically independent samples. ***P < 0.001, Statistical significance was assessed with one-way ANOVA with Tukey’s HSD test. **G**, **H** Statistical analysis of Egr1^+^ HMrSV5 cells (MFI). Bar charts shown data as mean values ± SD over *n* = 3 biologically independent samples. ****P < 0.0001. Statistical significance was assessed with two-tailed Student *t* test. **I** Immunoblot was used to determine the expressions of Vimentin, α-SMA, phosphorylated Smad3 and Smad3 in HMrSV5 cells with Egr1 overexpression or knockout. GAPDH was included as a loading control. **J**, **K** Smad luciferase reporter activity in HMrSV5 cells with Egr1 overexpression or knockout. Bar charts shown data as mean values ± SD over *n* = 3 biologically independent samples. **P < 0.01. Statistical significance was assessed with two-tailed Student *t* test. **L**, **M** Representative images of gel con*t*raction assay shown the effect of Egr1 on the ability of HMrSV5 cells to contract type I collagen in vitro (upper). Quantification of gel contraction assay from above experiment. Bar charts shown data as mean values ± SD over *n* = 3 biologically independent samples. **P < 0.01; ***P < 0.001, Statistical significance was assessed with two-tailed Student *t* test. **N**, **O** Representative images of chemotaxis assay shown the effect of HMrSV5-Egr1 cells on the migratory ability of NCI-N87 cells. The scale bar, 100 μm, 200× magnification. Statistical analysis of number of migratory cells. Bar charts shown data as mean values ± SD over *n* = 3 biologically independent samples. ****P < 0.0001. Statistical significance was assessed with two-tailed Student *t* test. **P**, **Q** Representative images of chemotaxis assay shown the effect of HMrSV5-KoEgr1 cells on the migratory ability of NCI-N87-HOXA11 cells and NCI-N87-Vector cells. The scale bar, 100 μm, 200× magnification. Statistical analysis of number of migratory cells. Bar charts shown data as mean values ± SD over *n* = 3 biologically independent samples. *P < 0.05; ***P < 0.001. Statistical significance was assessed with one-way ANOVA with Tukey’s HSD test. **R** The protein expression of CD133, CD44, CD90, N-cadherin and Vimentin in NCI-N87 cells co-cultured with HMrSV5 cells with Egr1 overexpression or knockout were analyzed using western blot with the indicated antibodies. GAPDH was included as a loading control. **S** Schematic diagram of the tumor-mesothelium HOXA11-PDGF BB/TGF β1-miR-181a-5p-Egr1 feedforward amplifier circuity.

Our data so far have proved that HOXA11-overexpressed GC cells upregulate the expression of Egr1 in HMrSV5 cells through forming a PDGF BB/TGF β1-miR-181a-5p feedforward loop with mesothelial cells, and prior studies found that Egr1 knockdown lessened liver fibrosis in a mouse model of thioacetamide-induced liver injury [26], moreover, Egr1 could favor alcohol induced steatosis [27] and human fibrotic disorders [9], however, there was no study focusing on the mechanism underlying Egr1 mediates mesothelial fibrosis and the effect of Egr1-overexpressed mesothelial cells on GC cells. To this end, we constructed Egr1-overexpressed HMrSV5 cells and knockout of Egr1 in HMrSV5 cells and validated the efficiency of transfection by western blotting, qRT-PCR and immunofluorescence analysis (Fig. 8A–H), notably, Egr1 could induce significant morphological changes in HMrSV5 cells, which exhibited spindle-like with a scattered distribution that was indicative of reduced cell-cell adhesion, while, Egr1 knockout reversed this phenomenon (Fig. 8C, D). Moreover, western blotting analysis revealed that Egr1 could upregulate the expression level of Vimentin and α-SMA and induce the phosphorylation of Smad3 in HMrSV5 cells, however, knockout of Egr1 exhibited the opposite effects on the expression level of Vimentin, α-SMA and the phosphorylation of Smad3 in HMrSV5 cells (Fig. 8I). To validate whether activation of Smad3 phosphorylation affects downstream transcriptional responses regulated by Egr1, Smad luciferase reporter plasmid was used to perform transient transfection assays and overexpression of Egr1 strongly prompted reporter activities, in contrast, knockout of Egr1 exhibited an opposite effect (Fig. 8J, K), furthermore, the gel contraction assay was performed and overexpression of Egr1 obviously promoted collagen-contracting ability of HMrSV5 cell in vitro, while, Egr1 knockout reversed this tendency (Fig. 8L, M). We have proved that HMrSV5 cells could enhance the migration of GC cells and upregulate the expression level of EMT and stem cell-like property markers (Fig. 3B, I), we next examined whether mesothelium Egr1 modulates the migration of GC cells. Indeed, Egr1 overexpressed HMrSV5 cells were able to propel migration of GC cells, while, knockout of Egr1 in HMrSV5 cells significantly thwarted the migration of HOXA11 overexpressed NCI-N87 cells (*P* < 0.001) (Fig. 8N–Q), most notably, the upregulation of EMT (N-cadherin and Vimentin) and stem cell-like property markers (CD133, CD44 and CD90) were identified in the GC cells co-cultured with HMrSV5-Egr1 cells compared with those co-cultured with HMrSV5-Vector cells, however, knockout of Egr1 in HMrSV5 cells exhibited the opposite effects on the expression level of above markers in the GC cells (Fig. 8R). Egr1, as a zinc finger transcription factor, could play its role by regulating the transcription of a wide array of downstream genes [28], and Smad4, as the co-Smad, could play a role in oligomerization and transportation of heteromeric Smad complex to nucleus where they could modulate gene transcription via DNA binding and protein-protein interactions [29]. Notably, prior study found that Egr1 could physically interact with Smad3 [30], to validate whether Egr1 could interact with Smad components in HMrSV5 cells, the confocal laser scanning microscope was used and we observed that Egr1 co-localized with Smad4 and overexpression of Egr1 increased Smad4 expression (Fig. S4a–c). Furthermore, co-immunoprecipitation (co-IP) was performed in HMrSV5-Egr1 cells and the control ones, Egr1 could upregulate the expression of Smad4 (Fig. S4d, “Input panels”), importantly, Egr1 was co-precipitated with Smad4 and Smad3 in HMrSV5 cells (Fig. S4d, “IP panels”, compare lanes 1, 2 and 3). Besides, RNA-sequencing analysis was performed to identify mRNAs and non-coding RNAs (circRNA, lncRNA and microRNA) that were significantly regulated by Egr1 (Figs. S4e, S5a–c and S6a–c). Principal component analysis (PCA) mapping with RNA-sequencing profiling data presented an obvious separation of samples into two groups corresponding to the HMrSV5-Egr1 cells and its counterparts (Fig. S6e). lncRNA or circRNA, acting as a competitive endogenous RNA (ceRNA) sponge, competitively interacted with miRNAs to modulate the derepression of miRNAs targets, and ceRNA regulatory networks were crucial in cancer development [31]. As shown in Fig. S6D, the Sankey plot presented the ceRNA regulatory networks consisting of the ncRNA and mRNA regulated by Egr1 which is predicted by bioinformation software. Furthermore, the function enrichment analysis was performed to explore the KEGG pathway and GO terms in which are involved, the regulated mRNA and regulated ncRNA target genes mainly involved in the biological processes, for example, “regulation of pathway-restricted SMAD protein phosphorylation”, “positive regulation of cell migration”, and “regulation of epithelial to mesenchymal transition” and KEGG pathways, such as “pathways in cancer” and “TGF-beta signaling pathway”. These enriched functions were mainly related to the function of Egr1, which is it mediates mesothelial fibrosis and prompts migration of GC cells.

## Discussion

Our work offers a promise for the understanding of a formerly ambiguous mechanism for peritoneal dissemination of GC that is related with HOXA11-PDGF BB/TGF β1-miR-181a-5p-Egr1 feedforward amplifier circuity (Fig. 8S).

Our work provides examples of the bidirectional interactions that happen between GC cells and the peritoneal mesothelial cells: GC cells promote mesothelial fibrosis, and the latter reciprocates by prompting migration of GC cells, thereby constructing a potentially self-amplifying positive feedback loop. This finding proposes that peritoneal mesothelial cells serve as an important and underappreciated component of the microenvironment in metastasis to peritoneum.

Mesenchyme transition of mesothelial cells had been suggested to be participated into the pathogenesis of peritoneal metastases [3], and mesothelial cells through MMT had been mentioned as a source of CAF [8, 32]. Antigen-presenting CAFs (apCAFs) are developed from mesothelial cells, which process activated by interleukin-1 and TGF-β, and apCAFs could stimulate regulatory T cells (Tregs) formation in pancreatic cancer, moreover, the process of mesothelial cells to apCAFs transition and Tregs formation induced by apCAFs could be inhibited by the antibody targeting mesothelin, which is one of the mesothelial cell markers [33]. We found Egr1 could stimulate the fibrosis of peritoneal mesothelial cells, and identified a strategy to inhibit the MMT of peritoneal mesothelial cells by BIBF.

Egr1 is also regarded as NEFI-A, Zif268, Krox-8, and TIS85 and is a transcription factor implicated in several essential physiological processes, such as cell development, growth and proliferation, and fibrosis and so on [34]. Egr1 has an activation regulatory region, repressive regulatory region, and DNA-binding domain consisting of three Cys2-His2 subclass zinc fingers that interact with the GC-rich consensus sequence [35]. Egr1 expression is rapidly activated by several extracellular signaling molecules, for example, growth factors, cytokines, and hormones, which could interact with the sequence of serum response elements in the promoter region of Egr1 [36]. Meanwhile, Egr1 is also expressed by cancer cells in multiple types of carcinoma, containing stomach cancer, breast cancer, head and neck cancer, uterine cervical cancer and ovarian cancer [34, 37–39], Egr1 could be upregulated by hepatocyte growth factor in hepatocellular carcinoma and gastric cancer, moreover, upregulated by epidermal growth factor in ovarian cancer, both of which lead to tumor cell metastasis [35, 40]. Egr1 could prompt EMT of non-small cell lung cancer (NSCLC) and gastric cancer cells and activate gastric cancer cells proliferation and invasion by stimulating β-catenin expression [35, 41, 42]. Besides, various kinds of cancer cells secrete extracellular vesicles to ameliorate the migration of vascular endothelial cells by launching the expression of Egr1 [35]. PDGF A could upregulate the expression level of Egr1 in skin adipocyte stem cell [43], PDGF BB could enhance the expression of *Egr1* mRNA and stimulate ROS/ERK/EGR1 pathways in vascular smooth muscle cells (VSMCs) [44], and TGF β1, the most potent pro-fibrotic cytokine, could enhance Egr1 expression in hepatic stellate cells (HSCs) [26]. In this study, PDGF BB and TGF β1, which released by HOXA11 high-expressed GC cells, could upregulate the expression of Egr1 in HMrSV5 cells.

A growing body of evidence demonstrated that TGF β1 pathway plays an important part in the reciprocal communication of GC cells and mesothelial cells [45]. TGF β1 could lead to receptors Smad 2/3 phosphorylation, and then the phosphorylated Smad2/3 are translocated into nucleus where they will bind to Smad4 to form a heteromeric complex, and the latter could serve as transcription factor to regulate gene expression [46]. In terms of fibroblasts, Egr1 is crucial for fibrotic responses to TGF β1 [47], due to TGF-β signaling being cell-type- and context-dependent, we proved that TGF β1 could induce Egr1 expression in HMrSV5 cells. A prior study had suggested that the effect of GC cells on mesothelial cells surpasses that of TGF-β1 alone, this implies that some other soluble factors released by GC cells might also affect mesothelial cell behavior [48], our work has identified that not only TGF β1 but also PDGF BB could stimulate the Egr1 expression and induce fibrosis in HMrSV5 cells.

Our study was limited to an in vitro transwell coculture system, other mechanisms that are independent of chemokines and cytokines might exist. Nonetheless, clearer delineation of the function of stromal Egr1 in peritoneal metastasis of GC should be explored through systemic Egr1 inhibition in genetically engineered mouse model of GC, and construction of conditional *Egr1*-null mice.

Specific therapeutic regimens tailored for peritoneal metastasis of GC were scarce, thus, this field is still in its infancy, we suggest that follow-up research into the prevention of fibrosis of peritoneal mesothelial cells could lead to enhance clinical management to inhibit the development of PM.

We hope our works will do favor to exploration of newer finding to promote management of peritoneal metastasis of GC, to improve prognosis for patients and to keep on dispelling nihilism.

## Conclusions

In conclusion, our study shed new light on Egr1 as a key driver that activates mesothelial fibrosis and prompts migration of GC cells, on the basis of our findings and works from other researchers, HOXA11-PDGF BB/TGF β1-miR-181a-5p-Egr1 feedforward amplifier circuity is proposed as an important mechanism that drives mesothelial fibrosis and prompts migration of GC cells. Thus, disrupting this circuity stands for a promising therapeutic strategy that might augment therapy in peritoneal metastasis of GC.

## Materials and methods

### Cell lines

The gastric cancer cell NCI-N87 and MGC-803 were purchased from ATCC, the human peritoneal mesothelial cell line HMrSV5 and gastric cancer cell SGC-7901 was obtained from the cell bank of the Chinese Academy of Sciences (Shanghai, China). All gastric cancer cell lines were cultured in Dulbecco’s modified Eagle’s medium (DMEM) supplemented with 10% fetal bovine serum (FBS), 100 U/ml penicillin, and 100 μg/ml streptomycin. HMrSV5 cells was cultured in Roswell Park Memorial Institute-1640 (RPMI-1640) medium supplemented with 10% FBS, 100 U/ml penicillin G, and 100 μg/ml streptomycin sulfate. Cells were cultured at a 37 °C incubator with 5% CO_2_. Cells were used for less than 180 days after receipt or resuscitation from cryopreservation.

### Tissue samples

Peritoneal metastatic lesions were obtained from patients who experienced a radical surgery of gastric cancer including resection of the peritoneum at the Department of Surgical Oncology, Ruijin Hospital. All GC patients provided informed consent to use their clinical and pathologic data. These experiments were approved by the Ethics Committee of Ruijin Hospital and executed in consistent with ethical principle of the World Medical Association Declaration of Helsinki.

### Plasmids transfection and virus infection

The expression vector encoding CMV-C-3FLAG-Egr1 was designed and synthesized by Genechem Incorporation (Shanghai). Synthetic miR-181a-5p mimics and inhibitor, miR-124-3p mimics, miR-183-5p mimics, miR-191-5p mimics and the appropriate negative controls were obtained from Genepharma Incorportation (Shanghai). The lentiviral vector encoding pWPXL- FLAG-HOXA11 was constructed by Genepharma Incorportation (Shanghai). The HOXA11 shRNAs (GCCATTGAGCCCGCCACTAAA and GCAGTCTCGTCCAATTTCTAT) were synthesized and then subcloned into the vectors (PGMLV-hU6-MCS-Puro-WPRE), respectively (Genomeditech Incorportation, Shanghai). Crispr guide RNA (gRNA) sequence design, synthetization and lentiviral packaging were constructed by Genepharma Incorportation (Shanghai), the sequence of target Egr1-gRNA were as follows: Forward: 5’-CTGCAGATCTCTGACCCGTT-3’; Backward: 5’-GTTGCTGCCGCTGCCCTCTG-3’. Empty lentiviral vector worked as the control. The efficiency of transfection was examined by Western blotting and qRT-PCR to assess protein and mRNA expression following cell collection.

For plasmid transfections, HMrSV5 cells were grown at 70% confluence in 24-well plates and stably transfected with plasmids using Lipofectamine 2000 (Cat.11668-019, Invtrogen, USA), following the protocols. After incubation for 24 h, the culture medium was replaced with a fresh medium, and the transfected HMrSV5-Egr1^+^ and HMrSV5-Vector cells were harvested after treatment with 10 μg/ml puromycin (Beyotime Biotechnology, Shanghai) for two weeks. The efficiency of transfection was examined by Western blotting and qRT-PCR to assess protein and mRNA expression.

For the transfection of miRNA mimics and inhibitor, HMrSV5 cells were grown at 70% confluence in 24-well plates and transfected with RNA oligoribonucleotides using Lipofectamine 2000 (Cat.11668-019, Invtrogen, USA), following the protocols. The transfected HMrSV5 cells were collected for the following experiments after 48 h. The efficiency of transfection was examined using qRT-PCR to evaluate miRNA expression.

For virus infection, the GC cell lines NCI-N87 and SGC-7901 were infected with the lentiviral vectors for HOXA11 overexpression in the presence of 10 mg/ml Polybrene, MGC803 cells were infected with the lentiviral vectors for HOXA11 knockdown in the presence of 10 mg/ml Polybrene, and HMrSV5 cells were infected with the lentiviral vectors for Egr1 knockout in the presence of 10 mg/ml Polybrene. After incubation for 24 h, the culture medium was replaced with a fresh medium, and the infected cells were harvested after treatment with 10 μg/ml puromycin (Beyotime Biotechnology, Shanghai) for two weeks. The efficiency of infection was examined by Western blotting and qRT-PCR to assess protein and mRNA expression.

### Western blotting

Total cell lysates were prepared using RIPA lysis buffer (New Cell & Molecular Biotech Co., Ltd, Soochow) containing phenylmethanesulfonyl fluoride (Beyotime Biotechnology, Shanghai) for 30 min on ice, and then the lysates were centrifuged at 16,000 × g, 4 °C for 10 min. Protein concentration was detected with Bio-Rad Protein Assay Kit (Bio-Rad) following the instructions. Lysates were separated by SDS-polyacrylamide gel electrophoresis followed by transferred onto a polyvinylidene difluoride membranes (Bio-Rad), and then membranes were blocked in 5% bovine serum albumin (BioShop) in TBST and probed with primary antibodies overnight in cold room. Secondary antibodies were incubated for 2 h at room temperature. The infrared imaging system (Tanon Life & Science Co., Ltd, Shanghai) and ECL substrate solution (Beyotime Biotechnology, Shanghai) were used for protein detection. The antibodies were listed in Table S1.

### RNA extraction and quantitative real-time PCR (qRT-PCR) analysis

RNA was obtained from the indicated cells using TRIzol reagent method (Invitrogen). Aliquots of total RNA were reverse transcribed into Complementary DNA following protocols and followed by mixing with primers, SYBR Green PCR MIX (Applied Biosystems) and germ-free water. All reactions were executed in triplicate and executed on an ABI Prism 7900HT sequence detection system (Applied Biosystems). The level of target genes was normalized to that of GAPDH (internal control) according to the comparative Ct methods.

For miRNA detection, miRNA first strand cDNA synthesis was performed as protocols (Sangon Biotech Incorporation) and qRT-PCR was performed using microRNAs qPCR Kit (Sangon Biotech Incorporation). All the reactions were run for three times and executed on an ABI Prism 7900HT sequence detection system (Applied Biosystems). The forward primers of miRNA and U6 small nuclear RNA (U6) were designed and synthesized by Sangon Biotech Incorporation, the common reverse primer was obtained from the same company. The U6 small nuclear RNA (U6) was applied as internal control for miRNA assays, the threshold cycle values were calculated by the comparative Ct method [49]. The primers used for qRT-PCR were listed in Table S2.

### Immunofluorescence staining and confocal scanning laser microscopy (CLSM)

The indicated HMrSV5 cells, NCI-N87-Vector cells and NCI-N87-HOXA11 cells co-cultured with HMrSV5 cells or alone were cultured on 8-well plates (Millipore, Mass, USA) and grew for 48 h. The cells were briefly washed with PBS, and then fixed in 4% formaldehyde for 30 min at room temperature, after fixation, the cells were washed with PBS (3 × 5 min) again, followed by permeabilizating with 0.1% Triton X-100 for 30 min and blocking using 5% BSA for 15 min. After that, the fixed cells or tissue sections were incubated with primary antibodies overnight at 4 °C atmosphere, and then incubated with species-specific secondary fluorescent antibodies in a light-proof environment for one hour at room temperature and DAPI was used to stain the nucleus in a light-proof environment at room temperature for ten min. Finally, the cells or tissue sections were washed with PBS (3 × 5 min), visualized, recorded, and analyzed using Carl Zeiss microscope, ZEN software (ZEISS Company) and Image J software (NIH, Md, USA). The antibodies were shown in Table S1.

The HMrSV5-Vector cells, HMrSV5-Egr1^+^ cells were cultured on 8-well plates (Millipore, Mass, USA) and grew for 48 h, respectively. The processes were as described above in the section of immunofluorescence staining. Confocal observation was performed using a Nikon Eclipse Ti (Nikon Solutions Co., Ltd, Tokyo, Japan) at excitation wavelengths of 405 nm (DAPI), 488 nm (FITC), and 561 nm (Cy3) and emission wavelengths of 417-477 nm (DAPI), 500–550 nm (FITC), and 570–1000 nm (Cy3). And the images were recorded and analyzed by Eclipse C2 (Nikon Solutions Co., Ltd, Tokyo, Japan) and Image J software (NIH, Md, USA). The antibodies were shown in Table S1.

### Immunohistochemistry

Immunohistochemistry staining of paraffin slices of mice’ peritoneal tumor tissues and GC patient’s peritoneal metastatic lesions which were carried out on 4 μm-thick slices were identified to be tumor by hematoxylin and eosin (H&E) staining, and then performed using EnvisionTM Detection System (Dako, Agilent Technologies, Ca, USA) as protocols. When antigen retrieval was executed, the slices were incubated with the primary antibodies overnight at 4 °C, and then incubated with the horseradish peroxidase labeled secondary antibodies at 37 °C for 30 min and finally all slides were observed with diaminobenzidine. The intensity of positive staining was measured with integrated optical density. The antibodies were listed in Table S1.

### Chromatin immunoprecipitation (ChIP)

ChIP assay was executed as protocols [1]. Briefly, SGC-7901 and NCI-N87 cells were collected in 15 ml tubes and fixed with 4 ml of 1% paraformaldehyde for 10 min at room temperature before being quenched with 1 ml of 1×glycine, respectively. Samples were rinsed with PBS twice and lysed with 1 ml of 1×lysis/wash buffer followed by sonicating to produce DNA fragments. After sonication, chromatin was pre-cleared and incubated with primary antibodies by rotation at 4°C overnight and then incubated with protein G agarose by rotation for additional 1 h. Immunoprecipitated DNA was eluted from the agarose and then DNA was reverse crosslinked by 5 M NaCl, RNase A, 0.5 M EDTA, 1 M Tris-HCL and Proteinase K and purification. DNA was obtained by phenol/chloroform extraction and ethanol precipitation. Primer sequences for ChIP-qPCR were listed in Table S2.

### Co-immunoprecipitation assay (Co-IP)

Co-IP assay was executed as described previously [1]. Briefly, HMrSV5-Vector and HMrSV5-Egr1^+^ lysates were lysed with IP lysis/Wash buffer, respectively. And then the Aliquots of lysates were pre-cleared with control agarose resin slurry by rotation at 4 °C for 4 h, subsequently the lysates were immunoprecipitated using appropriate antibodies or corresponding IgG control by rotation at 4 °C overnight. The beads were washed with elution buffer and boiled for 5 min in the next morning, the bound proteins were resolved using SDS-PAGE, followed by western blotting with corresponding antibodies.

### Luciferase reporter assay

Luciferase reporter assay about miRNA was executed as described previously [28]. Briefly, the amplified Egr1 3’UTR section and mutant section, which contained a substitution of six nucleotides (GAAUGU to CTTACA) within the miR-181a-5p binding site, were inserted into the pmiR-RB-REPORTTM vector and control vector (Genepharm, Shanghai), using the Xhol and NotI sites, respectively. Next, HMrSV5 cells were transfected with miR181a-5p mimics or miR-181a-5p inhibitor or vector, respectively. For the groups treated with co-cultured NCI-N87-HOXA11 cells or counterparts upon addition of neutralizing antibodies of PDGF BB and TGF β1 or BIBF. HMrSV5-Control cells or HMrSV5-miR-181a-5pi cells were treated with indicated concentration of neutralizing antibodies of PDGF BB and TGF β1 or BIBF for 24 h, meanwhile, NCI-N87-HOXA11 cells or counterparts were cultured into the upper compartments of the chamber with culture medium. After 48 h, the dual luciferase reporter system (Promega, USA) was applied to examined the luciferase activity.

Luciferase reporter assay about transcript factor was executed as described previously [1]. The wild type and truncating mutation of PDGF BB and TGF β1 were cloned into the pGL3-basic vector. the Smad luciferase reporter plasmid was designed and synthesized by YEASEN Incorporation (Shanghai). HMrSV5-Egr1^+^ cells, HMrSV5-Vector cells, HMrSV5-KoEgr1 cells and HMrSV5-Control cells were seeded in 24-well plate and co-transfected with Smad luciferase reporter plasmids and pRL-TK vector. For the groups treated with recombinant human PDGF BB or TGF β1 and neutralizing antibodies of PDGF BB or TGF β1, HMrSV5 cells were treated with indicated concentration of PDGF BB, TGF β1, neutralizing antibodies of PDGF BB or TGF β1 for 24 h. NCI-N87-HOXA11, NCI-N87-Vector, SGC-7901-HOXA11, SGC-7901-Vector, MGC803-Control, MGC803-shHOXA11#1 and MGC803-shHOXA11#2 cells were seeded in 24-well plate and co-transfected with the wild-type, truncating mutation PDGF BB or TGF β1 promoter luciferase reporter plasmids and pRL-TK vector. For the groups treated with recombinant human PDGF BB or TGF β1, NCI-N87 and SGC-7901 cells were added with indicated concentration of PDGF BB or TGF β1 for 24 h, and then cells were obtained and luciferase activity was illustrated using the Dual-Luciferase reporter assay system. The luciferase signal from the PDGF BB, TGF β1 and Smad reporters were normalized to the luciferase signal from the renilla reporter. Experiments were repeated in triplicated.

### Transwell chemotaxis assay

Human peritoneal mesothelial cells recruitment was testified using a transwell assay. HMrSV5 -vector, HMrSV5-Egr1^+^ cells, HMrSV5-Control and HMrSV5-Egr1^-^ (1 × 10^5^ cells) were seeded on the lower compartment of 24-well plate and were separated from the upper compartment by a 10 μm thick poly-carbon membrane with 8.0 μm pores. NCI-N87-HOXA11, NCI-N87-Vector, SGC-7901-HOXA11, SGC-7901-Vector, MGC803-Control, MGC803-shHOXA11#1 and MGC803-shHOXA11#2 cells (1 × 10^5^ cells) were placed on the upper compartment of the chamber with serum-free medium, respectively. After co-culturing for 24 h, chambers were washed using PBS buffer and GC cells were fixed in 1% paraformaldehyde and stained with 1% crystal violet. Non-migrated GC cells located at the upper chambers were cleared and washed using PBS buffer. Figures of five different location were captured. The ability of Human peritoneal mesothelial cells recruitment was evaluated by the number of migrated GC cells calculated per field respectively. Experiments were repeated in triplicated.

### ELISA assay

PDGF BB and TGF β1 content in the cell culture supernatant was detected by a PDGF BB ELISA Kit (Beyotime Biotechnology, Shanghai) and TGF β1 ELISA Kit (Beyotime Biotechnology, Shanghai) as protocols. For the groups treated with recombinant human PDGF BB or TGF β1, NCI-N87-HOXA11, SGC-7901-HOXA11, MGC803-shHOXA11, or HMrSV5 cells were added with indicated concentration of PDGF BB or TGF β1 for 24 h and then the medium was replaced with serum-free medium for another 24 h, finally, the cell culture supernatant was collected and detected for the concentration of PDGF BB and TGF β1. Experiments were repeated in triplicated.

### Gel contraction assay

Gel contraction assay was executed as described previously [50], briefly, HMrSV5-Egr1^+^, HMrSV5-Vector, HMrSV5-Control or HMrSV5-KoEgr1 cells (1 × 10^6^ cells) and 500 μl collagen suspension were mixed and seeded in a 24-well plate. The collagen gel was maintained at a 37 °C incubators until it had been polymerized. And then 1 ml of culture medium wea located atop each collagen gel lattice and gels were gently separated from the sides of well using the sterile spatula. The gels were imaged at day=0 and set as 100% and subtracting the percentage area that remained at 96 h. Experiments were repeated in triplicated. For the groups treated with recombinant human PDGF BB or TGF β1, neutralizing antibodies of PDGF BB or TGF β1, or BIBF, HMrSV5 cells were treated with indicated concentration of PDGF BB, TGF β1, neutralizing antibodies of PDGF BB, TGF β1 or BIBF (10 μM) for 24 h. For the groups co-cultured with GC cells, NCI-N87-HOXA11, SGC-7901-HOXA11 cells, or counterparts (1 × 10^5^ cells) were placed on the upper compartment of the chamber with culture medium and released from the lower compartment by a 10 μm thick poly-carbon membrane with 0.4 μm pores.

### Cytokine microarray analysis

Cytokine microarray assay was executed as described previously [51], the human cytokine antibody array was designed and produced by RayBiotech Incorporation. Membranes were incubated with serum-free culture medium collected from NCI-N87-HOXA11, SGC-7901-HOXA11 cells, and counterparts and processed according to manufacturer’s protocol.

### RNA-Sequencing analysis

HMrSV5 cells (1 × 10^5^ cells) in 6-well plates were co-cultured with NCI-N87-HOXA11, SGC-7901-HOXA11, counterparts or none for 96 h, respectively. HMrSV5 Egr1^+^ and HMrSV5 -Vector cells (1 × 10^5^ cells) were also seed in 6-well plates for 96 h. RNA was extracted from three biological replicates. RNA quality was examined by 2100 Bioanalyser (Agilent) and quantified using the ND-2000 (NanoDrop Technologies). All samples sent for library preparation and sequencing using the Illumina Hiseq4000 platform at Majorbio Biotech Co., Ltd. (Shanghai, China). Detection of circRNA, lncRNA and miRNA and analysis of different Gene expression were executed on the company’s cloud platform.

### Mouse tumor models

Peritoneal metastatic xenograft model was executed as described previously [1]. Briefly, HMrSV5 cells, NCI-N87-Vector cells and NCI-N87-HOXA11 cells were lentivirally infected with firely luciferase (FFLuc) fusion vector (Genepharma) and selected with 10 μg/ml puromycin. And then HMrSV5-FFLuc cells (0.25 × 10^5^ cells) and NCI-N87-Vector-FFLuc cells (1 × 10^5^ cells) or HMrSV5-FFLuc cells (0.25×10^5^ cells) and NCI-N87-HOXA11-FFLuc cells (1 × 10^5^ cells) mixed within 0.1 ml PBS buffer were injected into the abdomen of mice, respectively. After 4 weeks, Bioluminescence imaging (BLI) of luciferase activity was applied to record tumor mass and distribution in abdomen of mice with a Xenogen IVIS system under 2.5% isoflurane anaesthesia. Pictures of mice was executed by injection of D-luciferin 10 min before BLI and bioluminescence in peritoneal foci were calculated using Spectrum Living Image Software. Finally, mice were anaesthetized and killed for tissue retrieval.

Animal studies were performed as protocols approved by Department of Experimental Animal Science, School of Medicine, Shanghai Jiao Tong University. Mouse housing, husbandry, and care practices reached the minimum requirements set forth in the Animal Welfare Act and the Guide for the Care and Use of Laboratory Animal.

### Quantification and statistical analysis

Statistical analyses were calculated by statistical programming language R. The type and number of replicates, the statistical test used, and the test results were shown in the figure legends. An unpaired two-tailed Mann-Whitney *U*-test was performed for the comparison of two unpaired samples, and a two-tailed Student’s *t*-test was executed for the comparison of normally distributed parameters. ANOVA with Tukey’s post-test was applied for multiple comparisons which are grouped. Data were presented as mean and standard error unless specified otherwise. The level of significance in all graphs was shown as follows: *P < 0.05, **P < 0.01, ***P < 0.001, ****P < 0.0001. No randomization or investigator blinding approaches were performed during the experiments and data analysis.

### Supplementary information

Supplementary information

## Data Availability

The datasets used/or analyzed during the study are available from the corresponding author on reasonable request.
