# Supplementary material for: Tumor-mesothelium HOXA11-PDGF BB/TGF β1-miR-181a-5p-Egr1 feedforward amplifier circuity propels mesothelial fibrosis and peritoneal metastasis of gastric cancer
Source: Oncogene. 2023 Nov 21;43(3):171–88. doi: 10.1038/s41388-023-02891-4 (PMC10786717; doi:10.1038/s41388-023-02891-4)
Supplement: Supplementary file 1 — Supplementary information [file 41388_2023_2891_MOESM1_ESM.docx]

**Supplementary table1 Antibodies, sources, and dilution**

| Antibody | Source | Dilution (application) |
| --- | --- | --- |
| HOXA11 | Abcam; #AB54365 | 1:1000(WB) |
| CD44 | Cell Signaling Techinology; #3570 | 1:1000(WB)  1:500(IF) |
| CD133 | Abclonal; #A12711 | 1:1000(WB) |
| CD90 | Santa Cruz; #SC-19614 | 1:10000(WB) |
| Bmi1 | Abclonal; #A0211 | 1:1000(WB) |
| Sox2 | Abclonal; #A11501 | 1:1000(WB) |
| α-SMA | Abclonal; #AB5694 | 1:10000(WB)  1:200(WB) |
| Vimentin | Cell Signaling Techinology; #5741 | 1:1000(WB) |
| Ki67 | Abcam; #AB16667 | 1:250(IF) |
| Twist1 | Abclonal; #A3237 | 1:100(IF)  1:1000(WB) |
| Egr1 | Abclonal; #A2722 | 1:1000(WB)  1:100(IF) |
| Egr1 | Proteintech Group; #22008-1-AP | 1:100(IHC) |
| Egr1 | Cell Signaling Techinology; #4154 | 1:50(IP) |
| HBME-1 | DAKO; #M3505 | 1:50(IHC)  1:50(IF) |
| GAPDH-Mouse | Cell Signaling Techinology; #51332 | 1:1000(WB) |
| GAPDH-Rabbit | Cell Signaling Techinology; #5174 | 1:1000(WB) |
| DAPI | Beyotime; #C1002 | 1:1000(IF) |
| TGF β1 | Abcam; #AB92486 | 1:1000(WB) |
| PDGF BB | Abclonal; #A1195 | 1:1000(WB) |
| Cy3-Mouse | Proteintech Group; #SA00009-1 | 1:100(IF) |
| Cy3-Rabbit | Proteintech Group; #SA00009-2 | 1:100(IF) |
| TRITC Phalloidin | Yeasen; #40734ES75 | 1:100(IF) |
| Flag | Abclonal; #AE005 | 1:1000(WB)  1:50(ChIP) |
| Smad4 | Santa Cruz; #SC-7966 | 1:1000(WB)  1:100(IF) |
| Smad3 | Cell Signaling Techinology; #9513 | 1:1000(WB) |
| Phospho-Smad3(Ser423/425) | Cell Signaling Techinology; #9520 | 1:1000(WB) |
| Recombinant Human PDGF BB | R&D systems; 220-BB | 5 ng/mL |
| Recombinant Human TGF β1 | R&D systems; 7754-BH | 0.2 ng/mL |
| TGF-beta Pan Specific Antibody | R&D systems; AB-100-NA | 10 μg/mL |
| Human PDGF-BB Antibody | R&D systems; AF-220-NA | 0.5 μg/mL |

**Supplementary table 2 Primer sequences**

| Primer | Forward | Reverse |
| --- | --- | --- |
| GAPDH | GGACCTGACCTGCCGTCTAG | GTAGCCCAGGATGCCCTTGA |
| Egr1 | CTACTGGAGTGGAAGGTCTA | GAGGTGAAGAACTTGGACAT |
| Vimentin | AACTTAGGGGCGCTCTTGTCCC | CGCGCTGCTAGTTCTCAGTGCT |
| α-SMA | CTATGAGGGCTATGCCTTGCC | GCTCAGCAGTAGTAACGAAGGA |
| HOXA11 | TGCCAAGTTGTACTTACTACG  TC | GTTGGAGGAGTAGGAGTATGT  CA |
| PDGF BB | CAGCGCCCATTTTTCATTCC | TTTTCTCTTTGCAGCGAGGC |
| TGF β1 | GGCCAGATCCTGTCCAAGC | GTGGGTTTCCACCATTAGCAC |
| TGF β1-ChIP | CCCAGTTTCCCTATCTGTAAA | CCTGTAAGAATTGCTCTCCT |
| PDGF BB-ChIP | CCGAGGTACTGAGAGGTT | GAGACACAGCCTGAAGAC |
| U6 small nuclear RNA | CAAATTCGTGAAGCGTTCCATAT |  |
| miR-124-3p | TAAGGCACGCGGTGAATGC |  |
| miR-181a-5p | AACATTCAACGCTGTCGGTGAGT |  |
| miR-191-5p | CAACGGAATCCCAAAAGCAGCTG |  |
| miR-183-5p | CGCCTATGGCACTGGTAGAATTCACT |  |


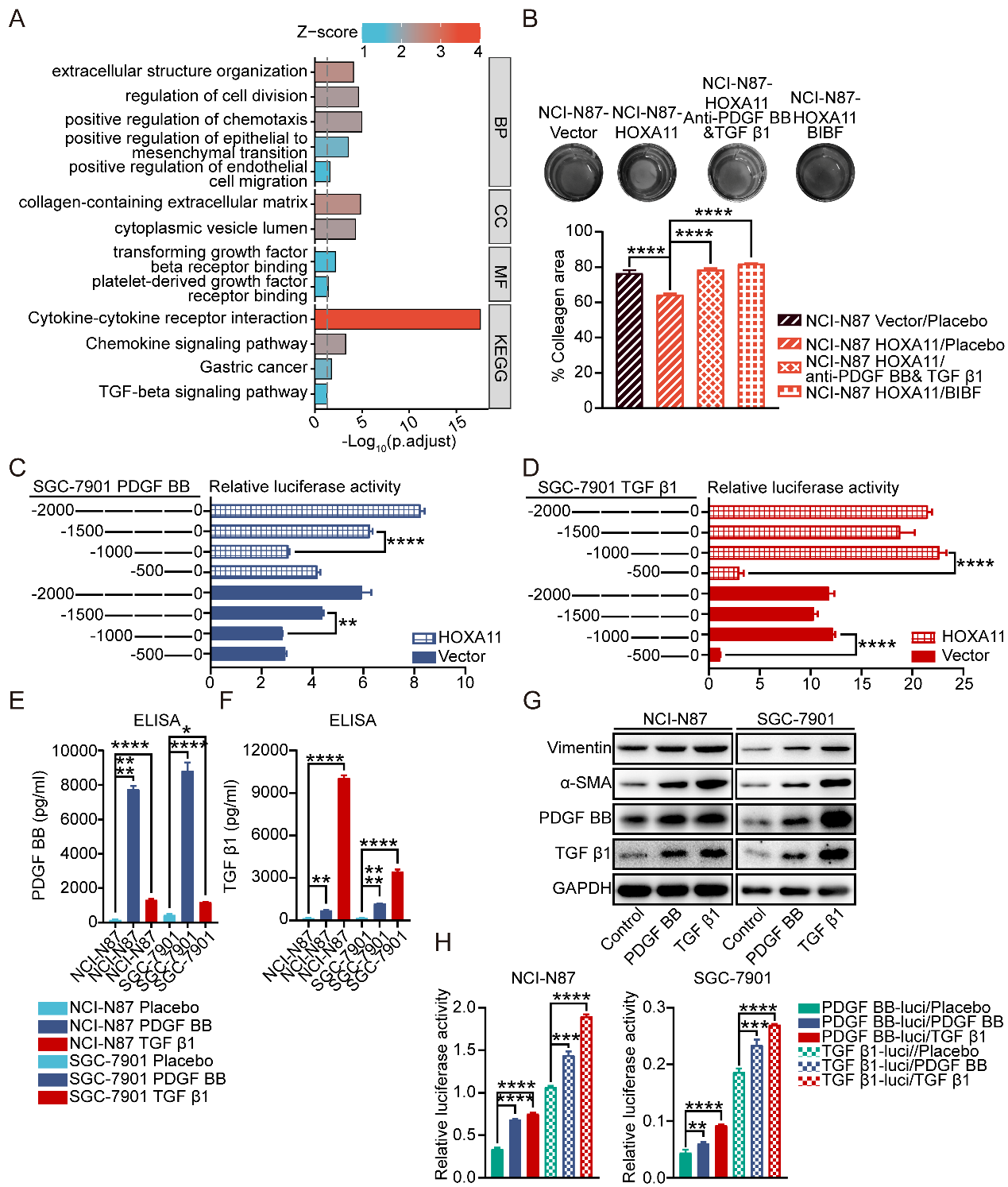


**Supplementary Fig.1 PDGF BB and TGF β1 transcriptionally regulated by HOXA11 and possessed autocrine loop in gastric cancer cells.** a. Bar chart represent the functional analysis of shared up-regulated and down-regulated chemokines/cytokines regulated by HOXA11 in NCI-N87 and SGC-7901 GC cells by GO enrichment and KEGG pathway analysis. b. Representative images of gel contraction assay shown the effect of co-cultured HOXA11 over-expressed gastric cancer cells on the ability of HMrSV5 cells to contract type I collagen *in vitro* upon neutralizing PDGF BB and TGF β1 or adding BIBF. Quantification of gel contraction assay from experiments. Bar charts shown data as mean values ± SD over n=3 biologically independent samples. ****, *P<*0.0001, Statistical significance was assessed with one-way ANOVA with Tukey’s HSD test. c&d. PDGF BB and TGF β1 luciferase reporter activity in SGC-7901 cells stably expressing HOXA11 or Vector with altered promoter zones. Bar charts shown data as mean values ± SD over n=3 biologically independent samples. **, *P<*0.01; ****, *P<*0.0001, Statistical significance was assessed with one-way ANOVA with Tukey’s HSD test. e. the density of PDGF BB in serum-free cultured medium collected from NCI-N87 cells and SGC-7901 cells after addition of PDGF BB, TGF β1, or placebo were measured using ELISA assay, respectively. Bar charts represent data as mean values ± SD over n=3 biologically independent samples. *, *P<*0.05; ****, *P<*0.0001, Statistical significance was assessed with one-way ANOVA with Tukey’s HSD test. f. the density of TGF β1 in serum-free cultured medium collected from NCI-N87 cells and SGC-7901 cells after addition of PDGF BB, TGF β1, or placebo were measured using ELISA assay, respectively. Bar charts represent data as mean values ± SD over n=3 biologically independent samples. **, *P<*0.01; ****, *P<*0.0001, Statistical significance was assessed with one-way ANOVA with Tukey’s HSD test. g. The protein expression of Vimentin, α-SMA, PDGF BB and TGF β1 in NCI-N87 cells and SGC-7901 cells after addition of PDGF BB, TGF β1, or placebo were analyzed using western blot with the indicated antibodies. GAPDH was included as a loading control. h. PDGF BB and TGF β1 luciferase reporter activity in NCI-N87 cells and SGC-7901 cells after addition of PDGF BB, TGF β1, or placebo, respectively. Bar charts shown data as mean values ± SD over n=3 biologically independent samples. **, *P<*0.01; ***, *P<*0.001; ****, *P<*0.0001, Statistical significance was assessed with one-way ANOVA with Tukey’s HSD test.


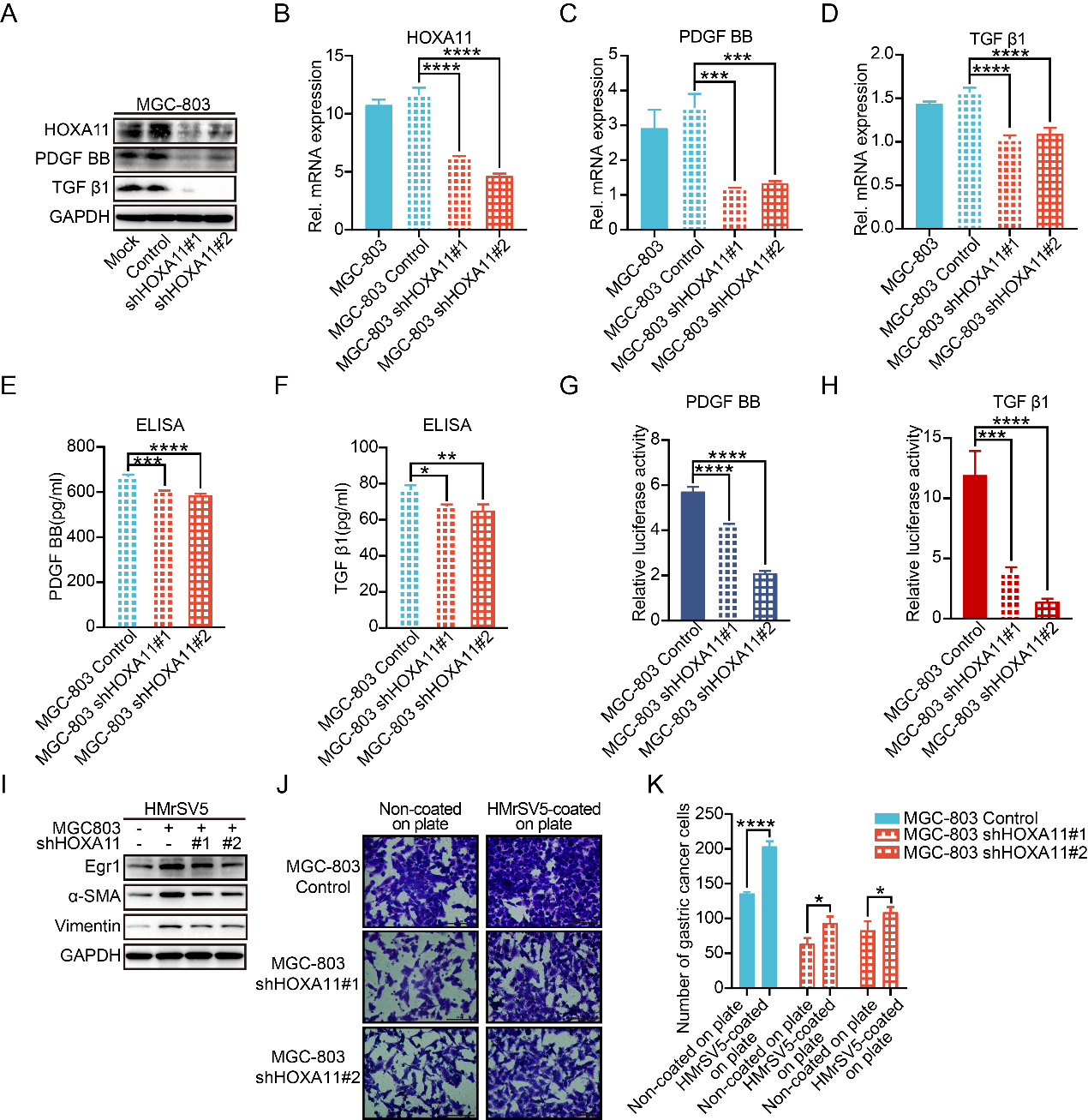


**Supplementary Fig.2 HOXA11 transcriptionally regulated PDGF BB and TGF β1 in gastric cancer cells and prompt the fibrosis of HMrSV5 cells.** a. Representative immunoblots of Control- and HOXA11 knocked-down MGC-803 cell lysates blotted as indicated, GAPDH was included as a loading control. b-d. qRT-PCR shown mRNA levels of the indicated genes in HOXA11 knocked-down MGC-803 cells and control cells. Bar charts shown data as mean values ± SD over n=3 biologically independent samples. ***, P<0.001; ****, P<0.0001, Statistical significance was assessed with one-way ANOVA with Tukey’s HSD test. e. The density of PDGF BB in serum-free cultured medium collected from HOXA11 knocked-down MGC-803 cells and control cells were measured using ELISA assay. Bar charts shown data as mean values ± SD over n=3 biologically independent samples. ***, P<0.001; ****, P<0.0001, statistical significance was assessed with one-way ANOVA with Tukey’s HSD test. f. the density of TGF β1 in serum-free cultured medium collected from HOXA11 knocked-down MGC-803 cells and control cells were measured using ELISA assay. Bar charts shown data as mean values ± SD over n=3 biologically independent samples. *, P<0.05; **, P<0.01, statistical significance was assessed with one-way ANOVA with Tukey’s HSD test. g&h. PDGF BB and TGF β1 luciferase reporter activity in HOXA11 knocked-down MGC-803 cells and control cells. Bar charts shown data as mean values ± SD over n=3 biologically independent samples. ***, P<0.001; ****, P<0.0001, Statistical significance was assessed with one-way ANOVA with Tukey’s HSD test. i. Expression of Egr1, Vimentin and α-SMA in indicated cells were analyzed using western blot, and GAPDH was applied as a loading control. j. Representative images of chemotaxis assay. The scale bar, 100 μm, 200× magnification. k. Statistical analysis of number of migratory cells. Bar charts shown data as mean values ± SD over n=3 biologically independent samples. *, P<0.05; ****, P<0.0001. Statistical significance was assessed with one-way ANOVA with Tukey’s HSD test.


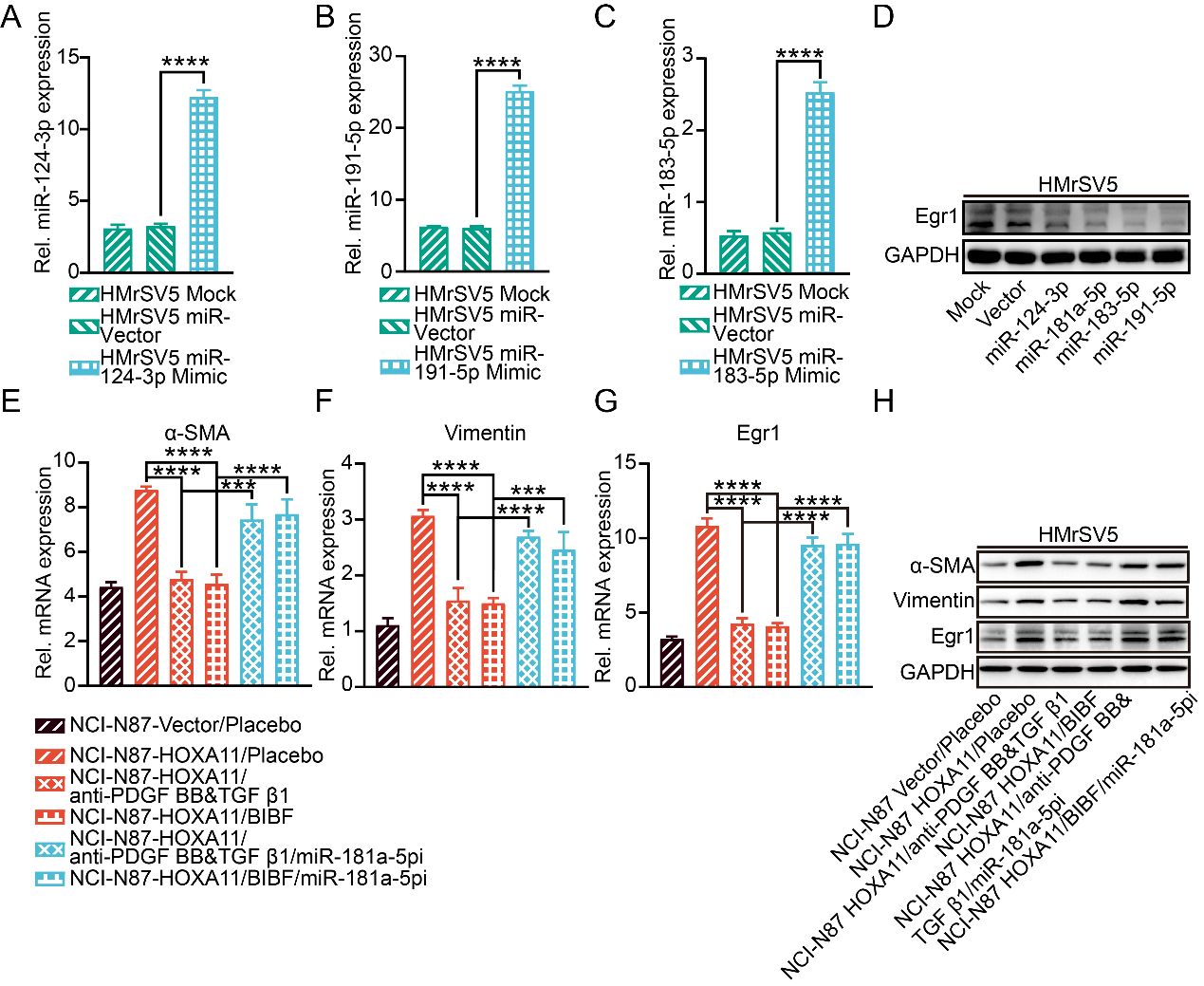


**Supplementary Fig.3 Gastric cancer cells-peritoneal mesothelial cells HOXA11-PDGF BB/TGF β1-miR-181a-5p feedforward amplifier circuitry drive fibrosis of peritoneal mesothelial cells.** a. qRT-PCR analysis of the expression level of miR-124-3p in HMrSV5 cells transfected with miR-124-3p mimic or Vector. The result was normalized to U6 small nuclear RNA. Bar charts shown data as mean values ± SD over n=3 biologically independent samples. ****, *P<*0.0001; Statistical significance was assessed with one-way ANOVA with Tukey’s HSD test. b. qRT-PCR analysis of the expression level of miR-191-5p in HMrSV5 cells transfected with miR-191-5p mimic or Vector. The result was normalized to U6 small nuclear RNA. Bar charts shown data as mean values ± SD over n=3 biologically independent samples. ****, *P<*0.0001; Statistical significance was assessed with one-way ANOVA with Tukey’s HSD test. c. qRT-PCR analysis of the expression level of miR-183-5p in HMrSV5 cells transfected with miR-183-5p mimic or Vector. The result was normalized to U6 small nuclear RNA. Bar charts shown data as mean values ± SD over n=3 biologically independent samples. ****, *P<*0.0001; Statistical significance was assessed with one-way ANOVA with Tukey’s HSD test. d. Representative immunoblots of Vector- and miR-124-3p, miR-181a-5p, miR-183-5p and miR-191-5p mimics transfected HMrSV5 cell lysates blotted as indicated, GAPDH was included as a loading control. e-g. qRT-PCR shown mRNA levels of the α-SMA, Vimentin and Egr1 in HMrSV5 cells regulated by HOXA11-PDGF BB/TGF β1-miR-181a-5p feedforward circuitry. Bar charts shown data as mean values ± SD over n=3 biologically independent samples. ***, *P<*0.001; ****, *P<*0.0001, Statistical significance was assessed with one-way ANOVA with Tukey’s HSD test. h. immunoblots shown the protein expression level of α-SMA, Vimentin and Egr1 in HMrSV5 cells regulated by HOXA11-PDGF BB/TGF β1-miR-181a-5p feedforward circuitry. GAPDH was included as a loading control.

**
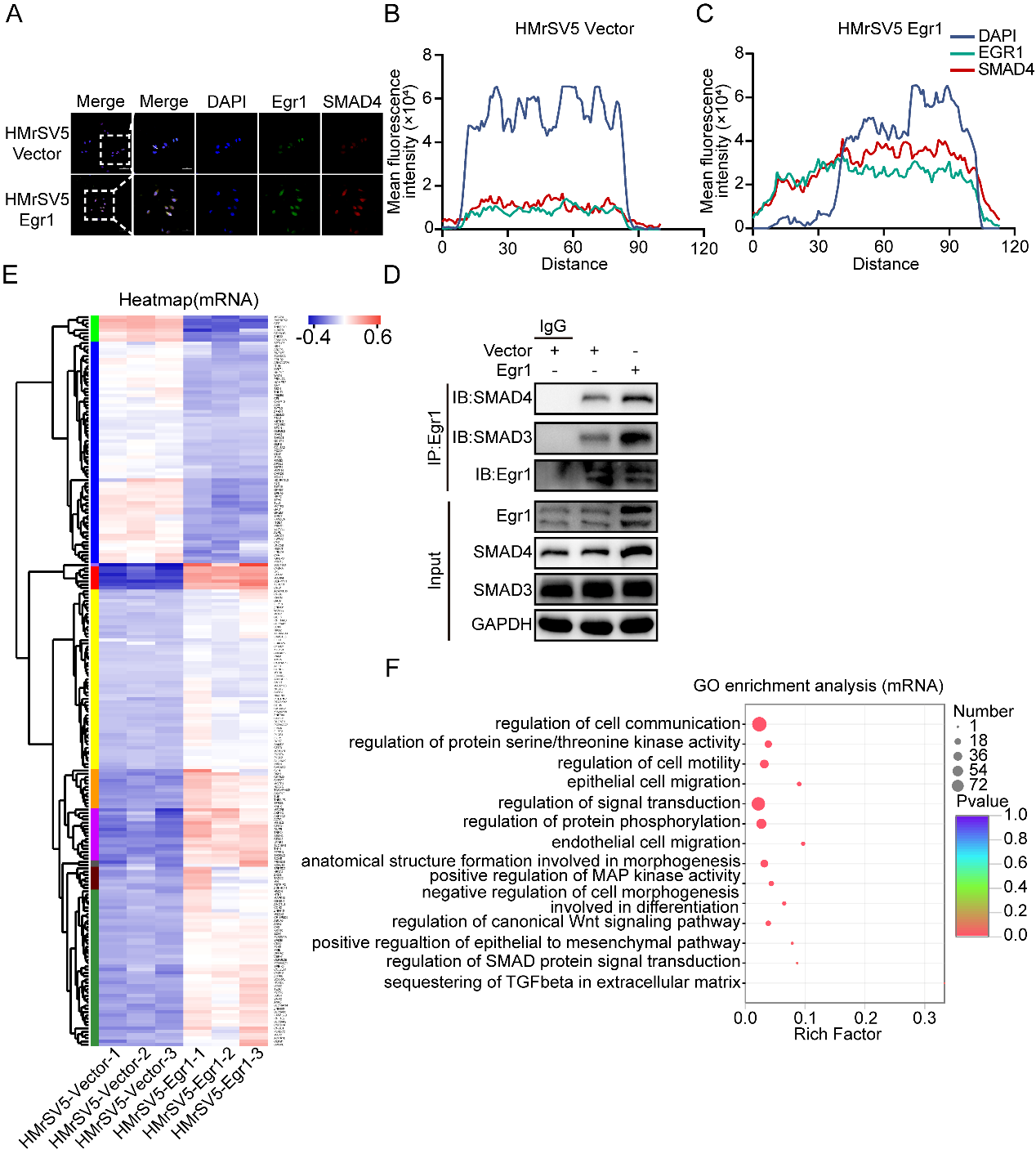
**

**Supplementary Fig.4 Physical interaction between Egr1, Smad3 and Smad4 in HMrSV5 cells.** a. Representative co-immunofluorescence images of subcellular localization of SMAD4 and Egr1 in HMrSV5 cells stably expressing Egr1 or Vector by confocal microscopy. The scale bar, 50 μm, 400× magnification. b&c. The line profiles of the mean fluorescence intensity of Egr1, Smad4 and DAPI signals in HMrSV5 cells stably expressing Egr1 or Vector were measured. d. Smad3 and Smad4 interacted with Egr1, determined by Co-IP experiments in the lysates of HMrSV5 cells stably expressing Egr1 or Vector using anti-Egr1 antibodies. e. Heatmap representation of the expression level of the 343 genes in HMrSV5 cells with over-expressed Egr1 and counterparts. f. bubble chart represented the functional analysis of up-regulated and down-regulated genes in HMrSV5 cells with over-expressed Egr1 and counterparts by GO enrichment.


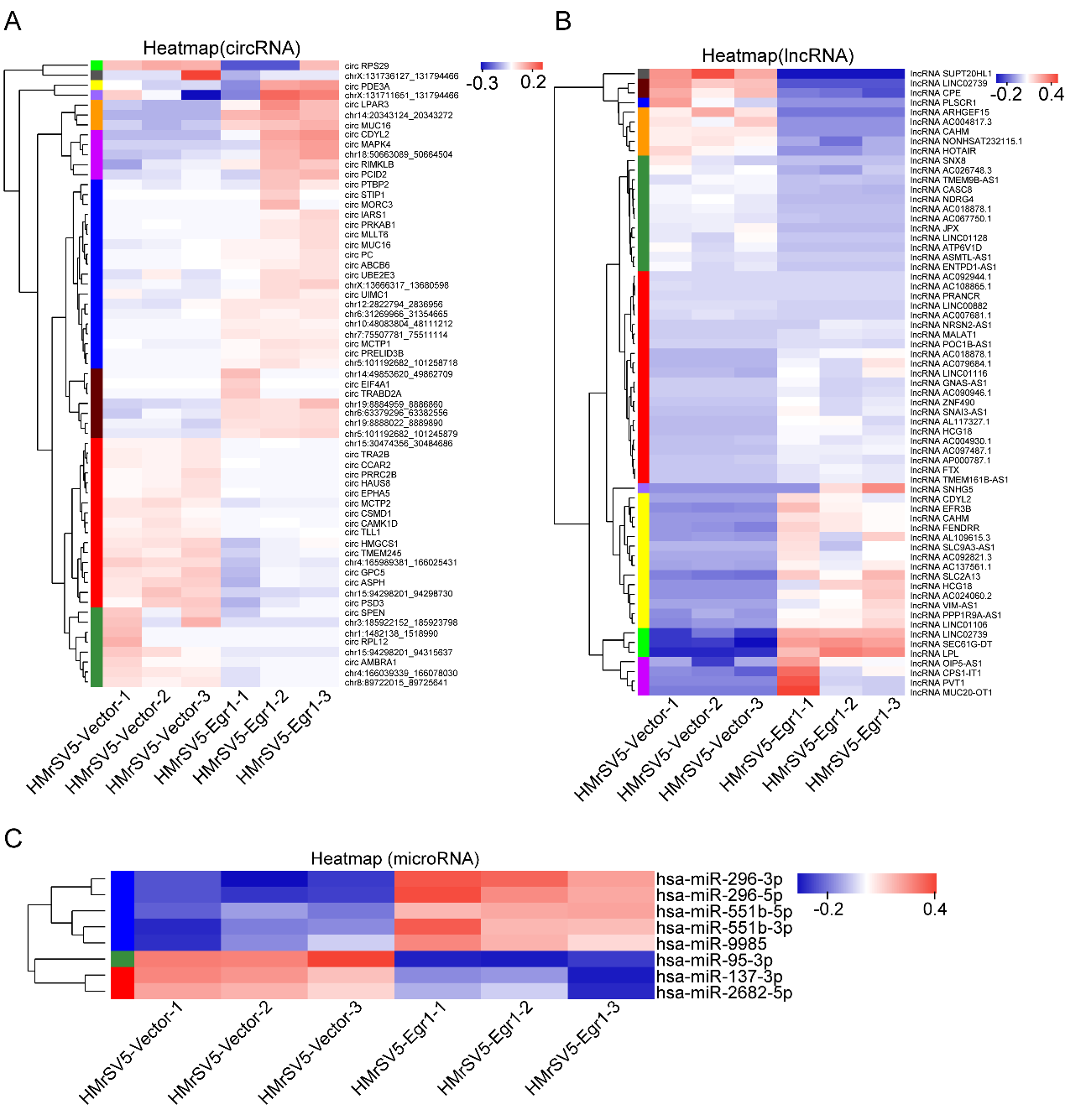


**Supplementary Fig.5 Egr1 modulated the expression of non-coding RNA in HMrSV5 cells.** a. Heatmap representation of the expression level of the 63 circRNA in HMrSV5 cells with over-expressed Egr1 and counterparts. b. Heatmap representation of the expression level of the 65 lncRNA in HMrSV5 cells with over-expressed Egr1 and counterparts. c. Heatmap representation of the expression level of the 8 miRNAs in HMrSV5 cells with over-expressed Egr1 and counterparts.


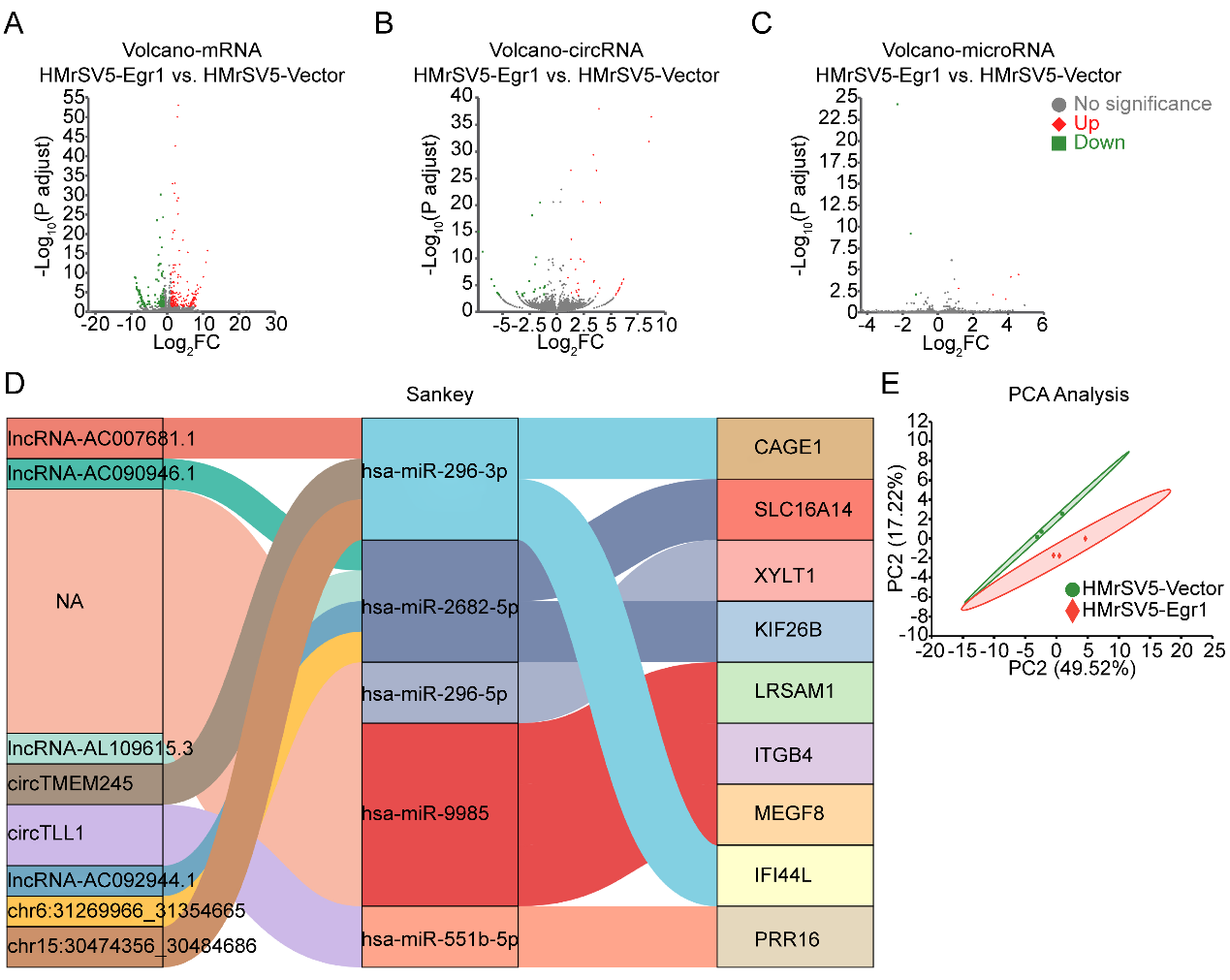


**Supplementary Fig.6 integrated analysis of lncRNA/circRNA-miRNA-mRNA ceRNA network regulated by Egr1 in HMrSV5 cells.** a. Volcano plots shown significantly changed genes in HMrSV5 cells with over-expressed Egr1 and counterparts. An absolute log2 fold-change >1 and an adjust P value <0.05 cutoff was applied to defined different expressed gene. b. Volcano plots shown significantly changed circRNA in HMrSV5 cells with over-expressed Egr1 and counterparts. An absolute log2 fold-change >1 and an adjust P value <0.05 cutoff was applied to defined different expressed circRNA. c. Volcano plots shown significantly changed microRNA in HMrSV5 cells with over-expressed Egr1 and counterparts. An absolute log2 fold-change >1 and an adjust P value <0.05 cutoff was applied to defined different expressed microRNA. d. Sankey diagram shown the ceRNA network of lncRNA/circRNA-miRNA-mRNA. e. The principal component analysis (PCA) of mRNA expression profiling data in HMrSV5-Egr1^+^ cells and HMrSV5-Vector. Axes depict principal component 1 (PC 1), and principal component 2 (PC 2).


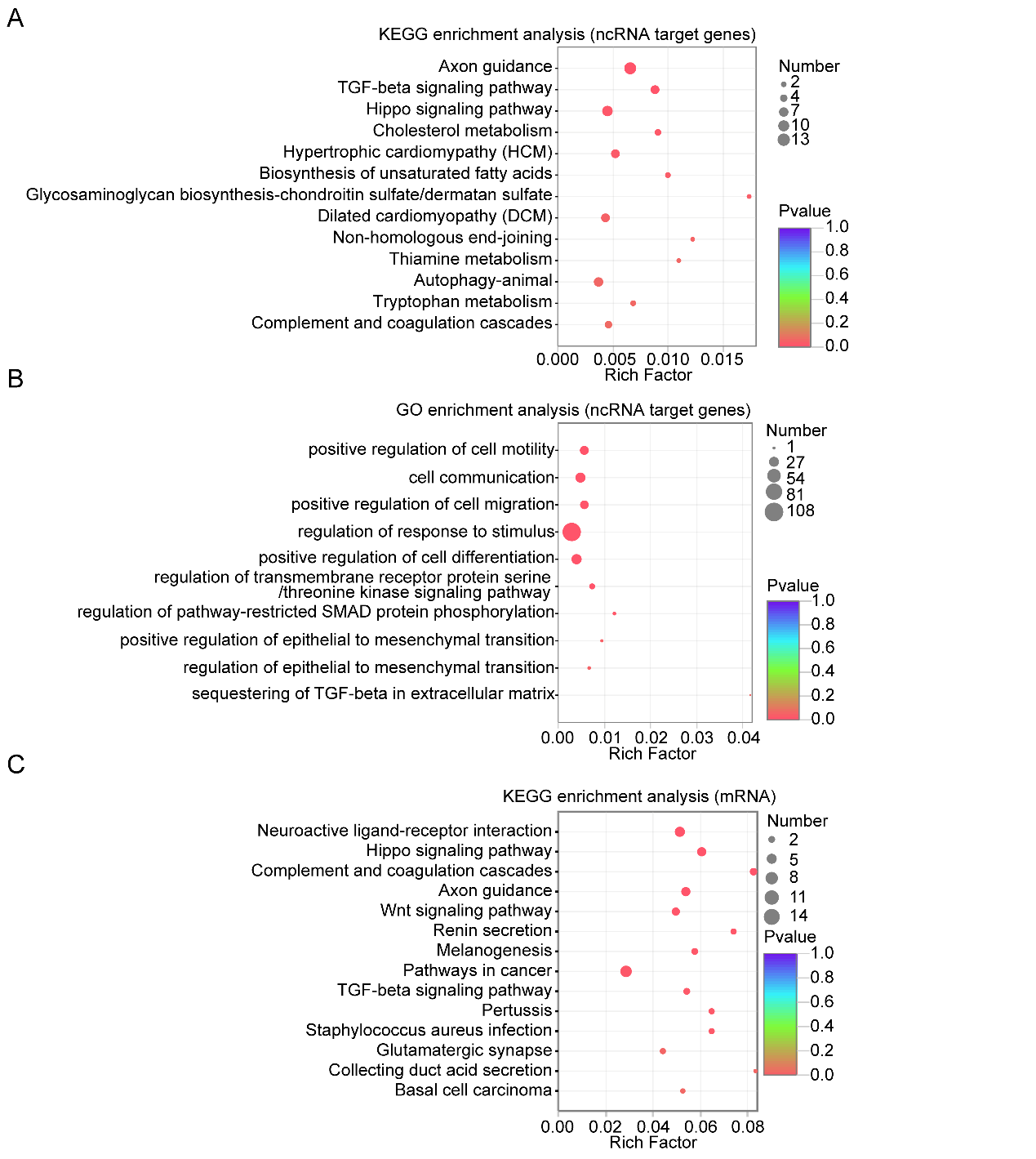


**Supplementary Fig.7 The functional analysis of ncRNA and mRNA regulated By Egr1 in HMrSV5 cells.** a. bubble chart shown the functional analysis of up-regulated and down-regulated ncRNA target genes in HMrSV5 cells with over-expressed Egr1 and counterparts by KEGG pathway analysis. b. bubble chart shown the functional analysis of up-regulated and down-regulated ncRNA target genes in HMrSV5 cells with over-expressed Egr1 and counterparts by GO terms analysis. GO terms contained biological process, cellular component, and molecular function. c. bubble chart shown the functional analysis of up-regulated and down-regulated genes in HMrSV5 cells with over-expressed Egr1 and counterparts by KEGG pathway analysis.
